# Sensory Adaptations to Improve Physiological and Behavioral Distress During Dental Visits in Autistic Children: A Randomized Crossover Trial

**DOI:** 10.1001/jamanetworkopen.2023.16346

**Published:** 2023-06-02

**Authors:** Leah I. Stein Duker, Dominique H. Como, Caitlin Jolette, Cheryl Vigen, Cynthia L. Gong, Marian E. Williams, José C. Polido, Lucía I. Floríndez-Cox, Sharon A. Cermak

**Affiliations:** 1 Division of Occupational Science and Occupational Therapy at the Ostrow School of Dentistry, University of Southern California, Los Angeles; 2 Current affiliation: Department of Occupational Therapy, School of Health and Rehabilitation Sciences, University of Pittsburgh, Pittsburgh, Pennsylvania; 3 Now with SOAR Autism Center, Denver, Colorado; 4 Fetal and Neonatal Institute, Division of Neonatology, Department of Pediatrics, Children’s Hospital Los Angeles, Los Angeles, California; 5 Keck School of Medicine, University of Southern California, Los Angeles, California; 6 University Center for Excellence in Developmental Disabilities, University of Southern California, Los Angeles; 7 Division of Dentistry, Children’s Hospital, Los Angeles, California; 8 Herman Ostrow School of Dentistry, University of Southern California, Los Angeles; 9 Now with Cedars-Sinai Medical Center, Los Angeles, California

## Abstract

**Importance:**

Autistic children have poorer oral health and greater oral care challenges, which are often associated with sensory overresponsivity, than neurotypical peers. It is important to identify innovative solutions enabling dentists to successfully perform standard clinic-based procedures for this population.

**Objective:**

To determine whether a sensory-adapted dental environment (SADE) reduces physiological and behavioral distress in autistic children undergoing dental cleanings, compared with a regular dental environment (RDE).

**Design, Setting, and Participants:**

This randomized crossover trial was conducted at a pediatric dentistry clinic in a large urban children’s hospital between May 2016 and April 2022. Coders were blinded to study condition for physiological but not behavioral measurements. Autistic children aged 6 to 12 years were identified and invited to participate. Interested families were enrolled consecutively; after confirmation of autism diagnosis, children were randomized. Analysis for this per-protocol study were conducted from April to October 2022.

**Intervention:**

Each child underwent 1 RDE and 1 SADE dental cleaning, administered in randomized and counterbalanced order approximately 6 months apart. SADE included modified visual, auditory, and tactile stimuli.

**Main Outcomes and Measures:**

The primary outcome was physiological stress, assessed by electrodermal activity. The secondary outcome was behavioral distress measured from video recordings.

**Results:**

Among 452 families invited to participate, 220 children were enrolled, and 162 children (mean [SD] age, 9.16 [1.99] years; 136 [84.0%] male) with confirmed autism were randomized, with 83 children receiving RDE first and 80 children receiving SADE first. Most children (94 children [58.0%]) had moderate autism severity. Children had significantly lower physiological stress during dental care in SADE compared with RDE (mean difference in skin conductance level, −1.22 [95% CI, −2.17 to −0.27] μS), suggesting decreased sympathetic activity and increased relaxation during SADE dental care. No significant differences were found in nonspecific skin conductance responses (mean difference, −0.30 [95% CI, −0.86 to 0.25] per min). Video-coded frequency and duration of behavioral distress (but not questionnaire) measures were significantly lower in SADE vs RDE (Cohen *d* = −0.84 to −1.19). Physiological stress was associated with behavioral distress during the dental cleaning (eg, nonspecific skin conductance responses associated with the Frankl Scale: β = −0.29; 95% CI, −0.39 to −0.19); age, IQ, and expressive communication moderated the intervention’s success. No participants withdrew due to adverse effects.

**Conclusions and Relevance:**

In this randomized crossover trial of autistic children, using SADE was safe and efficacious in decreasing physiological and behavioral distress during dental care. This is important because enhancing oral care is critical for autistic children; this intervention may also be beneficial for populations beyond autism.

**Trial Registration:**

ClinicalTrials.gov Identifier: NCT02430051

## Key Points

**Question:**

What is the effect of a sensory-adapted dental environment (SADE), compared with a regular dental environment (RDE), on the physiological stress of autistic children?

**Findings:**

In this randomized crossover trial including 162 autistic children, children exhibited significantly lower physiological stress in SADE compared with RDE in all phases of the dental visit, suggesting decreased sympathetic activity and increased relaxation during dental care in SADE.

**Meaning:**

These findings suggest that using a SADE during dental cleanings was a safe and efficacious way to improve dental experiences for autistic children.

## Teaser

This randomized crossover trial assesses physiological and behavioral distress in autistic children undergoing dental cleanings in a sensory-adapted dental environment compared with a regular dental environment.

## Introduction

Oral health, essential to overall health, is one of the most unmet health care needs for children in the United States.^1,2^ Children with special health care needs, such as autism, are at particular risk of oral health disparities.^3,4^ With the dramatic increase in autism prevalence (1 in 36 children diagnosed) over the past few decades,^5^ oral care in this population is a critical area of study.

Autistic children face numerous risks for poor oral health, including difficulties implementing home-based oral care practices^6,7,8^ and barriers accessing and tolerating in-office care^9,10,11,12^; both are associated with overresponsivity to sensory stimuli.^9,13,14,15^ Despite these challenges, there are minimal clinical dental protocols designed specifically to meet the needs of autistic pediatric patients^16,17^ and a reported lack of dental professionals willing and trained to serve the population.^9,10^

Attention to sensory sensitivities’ effect on oral care is becoming more prevalent in dentistry for autistic children, children with other disabilities,^9,13,18,19,20,21^ and neurotypical individuals.^22^ Shapiro and colleagues^23,24^ first proposed adapting the sensory characteristics of the dental office to reduce anxiety and enhance cooperation to facilitate oral care for children with developmental disabilities. Including the studies by Shapiro et al,^23,24^ several pilot studies have examined the preliminary efficacy of adapting the sensory environment of the dental office to improve care for individuals with special needs, with promising results.^20,25,26,27^ The purpose of this study was to conduct the first fully powered study, to our knowledge, examining the effect of a sensory-adapted dental environment (SADE), compared with a regular dental environment (RDE), on the physiological and behavioral distress of autistic children.

## Methods

### Study Overview

This clinical trial used a randomized crossover design. The study was approved by the institutional review board at Children’s Hospital Los Angeles (CHLA) and University of Southern California. All parents or guardians of participants provided informed consent, and participants provided assent when possible. Due to the presence or absence of modifications in the dental environment, blinding of treatment conditions for measures of behavioral distress were not possible. This study followed the Consolidated Standards of Reporting Trials (CONSORT) reporting guideline for randomized clinical trials. The trial protocol and statistical analysis plan are provided in Supplement 1.

### Recruitment and Enrollment

Child eligibility criteria were Spanish- or English-speaking, aged 6 to 12 years, at least 1 previous dental cleaning, and confirmed autism diagnosis. Exclusionary criteria included current or scheduled orthodontic braces; using anticholinergic medication; sibling of enrolled participant; genetic, endocrine, or metabolic dysfunction (eg, Down syndrome); significant motor impairment (eg, cerebral palsy); significant oral condition (eg, cleft palate); or medical condition placing the child at increased risk in the study (eg, uncontrolled seizures).

Families were recruited using a consecutive sampling strategy from an extensive network, including health clinics (eg, CHLA dental clinic), community service providers (eg, developmental disability service providers, resource fairs), therapy and behavioral clinics, patient referrals, parent support groups, social media, and the Los Angeles Unified School District.

### Procedures

#### Enrollment and Eligibility

Visit 1 took place at a location convenient to the family (eg, home, local library); participants were consented, assented when appropriate, and child-descriptor measures were completed. Visit 2 included confirmation of autism diagnosis and IQ assessment at CHLA. Following diagnosis confirmation, participants were randomized (stratified by sex and age [6.0-9.5 years and 9.6-12.11 years]) to order of treatment (eMethods in Supplement 2).

#### Intervention Dental Visits

Children participated in 2 routine dental cleanings approximately 6 months apart. Both cleanings were performed in a standard manner (oral examination, prophylaxis, fluoride) by second-year residents of a 2-year advanced pediatric dentistry program (provides didactic and clinical training required for the specialty) in the same private dental clinic room. Approximately 1 to 2 weeks before each dental visit, a tailored (eg, child sex, language, dental visit condition) social story was provided to participants; parents read it to their child several times to familiarize them with the upcoming dental visit (a copy may be requested from L.I.S.D).

The RDE (control) condition included no modifications. The SADE (experimental) condition included sensory-based modifications based on principles of Ayres sensory integration theory^28,29^ and multisensory environments.^30^ For the visual sensory domain, all overhead fluorescent lights and the dental operatory lamp were turned off and darkening curtains were applied to windows. A glasses-mounted headlight directed light into the child’s mouth (avoiding eyes), and slow-moving visual effects (ie, bubbles or fish scenes chosen by the child or parent) were projected onto the ceiling in the child’s visual field. For the auditory sensory domain, a playlist of calming music (eg, classical music with nature sounds) played in the room via a small speaker. Finally, for the tactile and deep pressure domain, a butterfly-shaped wrap,^26^ weighted with a pediatric dental radiograph vest was used to apply deep tactile pressure stimuli to the child. The butterfly wrap fit around the dental chair, with the wings wrapping around the child from shoulders to ankles to provide a calming deep hug sensation.^31,32^ While all SADE modifications were intended to be calming, if the child or parent felt uncomfortable about some aspect of the procedure, study protocol dictated that it would be discontinued on request. An implementation checklist was used for both dental conditions to ensure fidelity.

Dental cleanings began in July 2016 and ended in April 2022; due to COVID-19 precautions, data collection was suspended March 2020 to January 2021. No changes in dental care protocols occurred during the study period. We examined visits before vs after COVID-19 as a potentially moderating variable.

### Measures

#### Child Descriptor Measures

Demographic information (eg, child sex, age, race and ethnicity) was parent-reported via questionnaire as Asian, Black, American Indian, Pacific Islander, White, or more than 1 race; ethnicity was reported as Hispanic or non-Hispanic. Reporting race and ethnicity, mandated by the US National Institutes of Health (NIH), was consistent with the Inclusion of Women, Minorities, and Children policy. Communication ability, cognitive ability, general anxiety, dental anxiety, and sensory overresponsivity were collected; autism diagnosis was confirmed and severity score calculated (eMethods in Supplement 2).

### Outcome Measures

#### Physiological Stress and Anxiety

Electrodermal activity (EDA), the primary outcome, measured eccrine sweat gland activity produced by sympathetic nervous system activation. EDA was measured continuously using a MP150 System (BIOPAC) during a 3-minute baseline period immediately preceding care and throughout the entire dental cleaning, using pregelled electrodes on the distal phalanges of the index and middle fingers of the participant’s nondominant hand, a traditional method of EDA data collection.^33^ Tonic skin conductance level (SCL) and frequency per minute of nonspecific skin conductance responses (NS-SCRs) were collected, as these measures increase in stressful or anxiety-producing situations and are best suited for longer-duration situations.^33^

#### Overt Behavioral Distress

Each child was video recorded during each dental cleaning. The Children’s Dental Behavior Rating Scale (CDBRS),^26^ developed by our team and strongly correlated with traditional measures of uncooperative behavior (*r* ≥ 0.82),^34^ coded the presence or absence of 3 distress behaviors (ie, mouth, head, and forehead movement) and severity of whimper, cry, or scream and verbal stall or delay each minute of a 5-minute videotape of prophylaxis. Summed raw scores (range, 0-45) were converted to scale scores of 1 to 100 via Rasch analysis; higher scores indicated greater distress. A subset of 16% of videos were double-coded with at least 85% agreement; coding discrepancies were resolved through discussion until consensus was reached.

Similar to other studies examining the efficacy of SADE,^23,24,25^ frequency and duration of distress behaviors were also scored from video recordings. Count of mouth and head movements and count and duration of verbal distress behaviors (ie, whimper, cry, or scream) were coded from a 5-minute videotape of prophylaxis. A subset of 20% of videos were double-coded with at least 85% agreement; coding discrepancies were resolved through discussion until consensus was reached.

### Additional Measures

Child-reported measures of pain and sensory discomfort were collected following each dental cleaning. In addition, 2 measures of child behavior were obtained from dentists immediately after each dental cleaning. All are described in the eMethods in Supplement 2.

### Statistical Analysis

Power analysis and statistical analyses details are provided in the eMethods in Supplement 2. Although this study did not exhibit excessive loss to follow-up, we recognize the effect missing data can have on results and that even robust statistical methods cannot account for bias introduced by data not-missing-at-random. Therefore, multiple analytic methods were used to ensure that results did not differ materially by method. Methods included paired-sample *t* tests, Wilcoxon signed-rank tests, linear mixed-effects regression, and linear mixed-effects regression with square root transformation, with all methods fully recognizing the correlation between within-individual observations. To account for missing data, mixed-effects regression models based on restricted maximum likelihood estimation (generally considered to be superior to multiple imputation in accounting for missing data) were used.^35,36^

For the primary outcome, mean EDA scores, blindly assessed for both SCL and NS-SCRs, were calculated for the oral examination, prophylaxis, and fluoride stages for each child at each dental visit (eMethods in Supplement 2).

For secondary end points, Wilcoxon signed-rank tests and mixed-effects regression with square root transformation were used for EDA variables. Behavioral variables were not square-root transformed.

#### Mediation Analyses

We proposed to test the hypothesis that a favorable difference in secondary outcomes (distress behavior, pain, sensory discomfort) between SADE and RDE was mediated by physiological stress (SCL or NS-SCR frequency). For physiological stress to mediate the difference between SADE and RDE outcomes, outcomes would need to differ for SADE vs RDE, and physiological stress would need to be associated with both SADE and RDE and the outcome. If there were no significant differences between SADE and RDE for most of the secondary outcomes (pain, sensory discomfort, behavioral distress measured by CDBRS), there would be no mediation of these outcomes. Nevertheless, understanding whether lack of association between the treatment and those outcomes was due to lack of association between the treatment and the mediators or lack of association between the mediators and the outcomes would be informative; such analyses used mixed-effects regression with adjustment for attained age and first and second clinic visit, with EDA values square-root transformed (eMethods in Supplement 2).

#### Moderation Analyses

The dependent variables were values for each child at each dental visit for mean EDA for means and frequencies (primary outcomes) and video-coded values for distress behavior frequency and duration. Potential effect moderators were dichotomized either at their medians or a clinically meaningful point. Stratified least-squares means and SEs by RDE and SADE and by dichotomized potential moderators were calculated. An interaction term for the potential moderator with SADE and RDE was added to the mixed effects regression model to test the significance of effect moderation (eMethods in Supplement 2).

Data were analyzed using SAS statistical software version 9.4 (SAS Institute). *P* values were 2-sided, and statistical significance was set at *P* = .05. Data were analyzed from April to October 2022.

## Results

**Figure 1.**
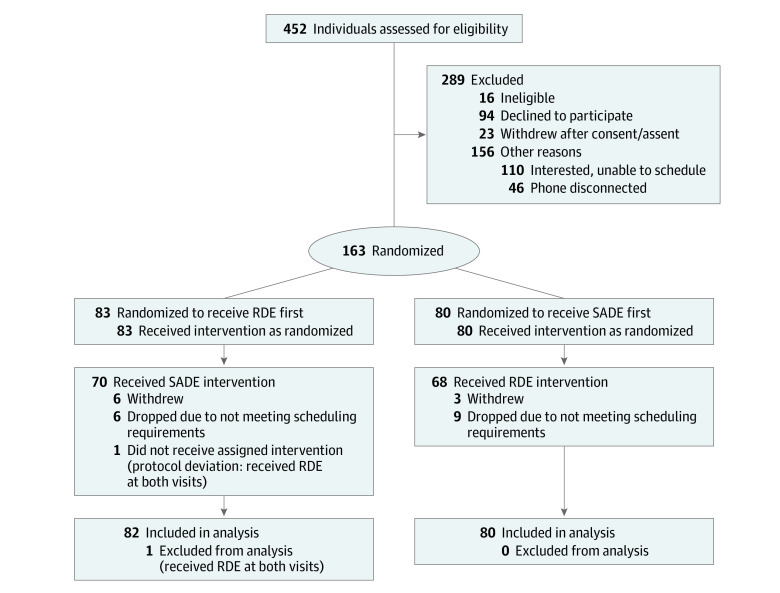
Participant Recruitment Flowchart

Of the 220 children enrolled in the study, 163 met eligibility criteria and received at least 1 dental cleaning, while 139 completed all study activities (Figure 1). One child received the same condition twice (protocol deviation) and removed from the data set; therefore, 162 children received their first dental cleaning and 138 children received their second. Participants were a mean (SD) age of 9.16 (1.99) years, and most participants (136 participants [84.0%]) were male. There were 13 Asian participants (8.0%), 14 Black participants (8.6%), no American Indian participants, 1 Pacific Islander participant (0.6%), 124 White participants (76.5%), and 10 participants (6.2%) identified as more than 1 race; 117 participants (72.2%) were Hispanic and 45 participants (27.8%) were non-Hispanic. Most children (94 children [58.0%]) had moderate autism severity (eTable 1 in Supplement 2). Most of the missing data in this study were due to participants not attending the second dental visit. Children who completed only 1 dental cleaning were demographically similar to those who completed 2 cleanings; only sensory overresponsivity was significantly different between the groups, with parents of the children receiving only 1 cleaning reporting significantly more overresponsivity compared with those completing both dental cleanings (mean [SD] SensOR score, 34.08 [15.48] vs 27.38 [13.54]; *P* = .03) (eTable 1 in Supplement 2). No significant demographic differences were found between the children who completed 2 dental cleanings stratified by order of intervention condition (Table 1).

**Table 1.** Participant Characteristics by Intervention Condition Order

Characteristic	Participants, No. (%)	
	RDE-first (N = 70)	SADE-first (N = 68)
Age, mean (SD), y		
At first eligibility visit	9.19 (2.04)	9.33 (1.93)
At first dental visit	9.73 (2.12)	9.77 (1.96)
At second dental visit	10.47 (2.15)	10.52 (1.99)
Time between dental visits, mean (SD), mo	8.95 (4.36)	8.98 (5.87)
Sex		
Male	58 (82.9)	56 (82.4)
Female	12 (17.1)	12 (17.6)
Race		
Asian	4 (5.7)	8 (11.8)
Black	7 (10.0)	4 (5.9)
American Indian	0	0
Pacific Islander	0	1 (1.5)
White	53 (75.7)	52 (76.5)
>1	6 (8.6)	3 (4.4)
Ethnicity		
Hispanic	50 (71.4)	47 (69.1)
Non-Hispanic	20 (28.6)	21 (30.9)
Diagnoses		
ADHD	16 (22.9)	10 (14.7)
≥1 Other diagnoses^a^	27 (38.6)	27 (39.7)
ADOS-2 severity score, mean (SD)	6.19 (1.78)	6.65 (1.89)
Low (3-4)	16 (22.9)	12 (17.6)
Moderate (5-7)	42 (60.0)	37 (54.4)
High (8-10)	12 (17.1)	19 (27.9)
WASI-II FSIQ4, mean (SD)^b^	74.71 (23.14)	71.56 (24.40)
<70	32 (45.7)	37 (54.4)
≥70	38 (54.3)	31 (45.6)
VABS-II Expressive Communication, mean (SD)^c^	64.97 (29.15)	63.63 (27.29)
<83	43 (61.4)	44 (64.7)
≥83	26 (37.1)	24 (35.3)
NA	1 (1.4)	0
SensOR Inventory Total, mean (SD)^d^	25.36 (12.95)	29.46 (13.91)
CASI-4 Autism Anxiety Scale, mean (SD)^e^	18.55 (9.91)	21.39 (10.47)
CFSS-DS, mean (SD)	45.94 (14.92)	49.25 (12.10)
No fear (<32)	13 (18.6)	4 (5.9)
Borderline fear (32-38)	12 (17.1)	11 (16.2)
High fear (>38)	45 (64.3)	53 (77.9)
Mother’s education		
<High school	8 (11.4)	12 (17.6)
High school	12 (17.1)	10 (14.7)
Some college or vocational	28 (40.0)	27 (39.7)
College degree	22 (31.4)	19 (27.9)
Father’s education		
<High school	7 (11.1)	14 (24.1)
High school	21 (33.3)	15 (25.9)
Some college or vocational	18 (28.6)	13 (22.4)
College degree	17 (27.0)	16 (27.6)

Abbreviations: ADOS-2, Autism Diagnostic Observation Schedule-2; CASI-4, Child and Adolescent Symptom Inventory, 4th edition, Autism Spectrum Disorder Anxiety Scale; CFSS-DS, Children’s Fear Survey Schedule–Dental Subscale; VABS-II, Vineland Adaptive Behavior Scales-II Expressive Communication; WASI-II FSIQ4, Wechsler Intelligence Scale Full Scale Intelligence Quotient.

^a^ Other diagnoses available for selection on survey and number endorsed within the full analytic cohort of 162 children: 1 child with learning disability, 4 children with developmental disability, 2 children with dyslexia, 4 children with sensory integration disorder, 4 children with epilepsy, 7 children with anxiety disorder.

^b^ IQ score of 70 is used to determine intellectual disability.

^c^ Range, 0 to 108; raw score of 83 is equivalent to the expressive language of a child aged 4 years.

^d^ Range, 0 to 76; higher score indicates greater over-responsivity to common sensory experiences.

^e^ Range, 0-60; greater anxiety indicated by higher score.

### Intervention Fidelity

Of 150 participants who underwent a cleaning in the SADE, more than 90% used the projector (147 children [98.0%]), music (145 children [96.7%]), and headlamp and lights off (135 children [90.0%]). A total of 112 children (74.7%) used the butterfly wrap.

### Primary Outcome

**Figure 2.**
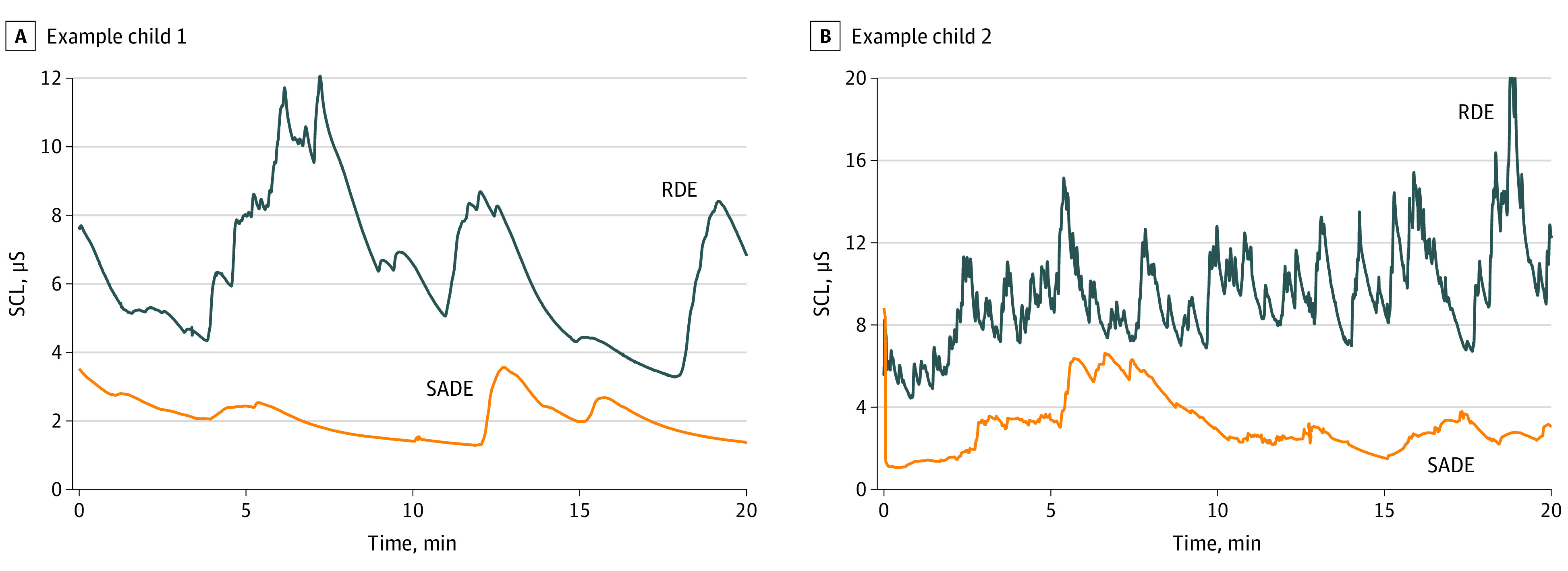
Electrodermal Activity of the First 20 Minutes of Dental Cleanings in the Regular Dental Environment (RDE) and Sensory-Adapted Dental Environment (SADE) in 2 Autistic Children Raw electrodermal activity data are presented. SCL indicates skin conductance level.

Overall, SCL was significantly lower in participants in the SADE condition than those in the RDE condition (mean difference of SCL, −1.22 [95% CI, −2.17 to −0.27] μS) (Table 2 and Figure 2). These results were robust regardless of statistical analysis method (Table 2). Significant differences were present throughout all 4 phases of the dental encounter (previsit rest, oral examination, prophylaxis, and fluoride) (Table 3).

**Table 2.** Primary Outcomes Evaluated with Different Statistical Methods

Measure	EDA: SCL^a^		EDA: NS-SCR frequency^b^		
	μS (95% CI)	*P* value	No. per min	*P* value
Mean (SD)				
SADE	7.74 (4.85)	NA	3.90 (2.72)	NA
RDE	8.96 (5.29)	NA	4.25 (3.03)	NA
Mean difference^c^	−1.22 (−2.17 to −0.27)	.01	−0.30 (−0.86 to 0.25)	.28
Median difference^d^	−1.34 (−2.92 to −0.26)	.01	−0.24 (−0.57 to 0.51)	.39
β for SADE vs RDE^e^	−1.20 (−2.12 to −0.27)	.01	−0.31 (−0.84 to 0.22)	.25
β for SADE vs RDE^f^	−0.21 (−0.39 to −0.04)	.02	−0.08 (−0.22 to 0.06)	.26

Abbreviations: EDA, electrodermal activity; NA, not applicable; NS-SCR, nonspecific skin conductance responses; RDE, regular dental environment; SADE, sensory-adapted dental environment; SCL, skin conductance level.

^a^ Including data from 121 SADE visits and 127 in the RDE visits.

^b^ Including data from 120 SADE visits and 125 RDE visits.

^c^ One-sample *t* test of (score with SADE) − (score with RDE). The full analytical sample used 106 observations for EDA mean and 105 observations for EDA frequency.

^d^ Wilcoxon signed rank test.

^e^ Mixed effects regression model with adjustment for attained age and first or second clinic visit. The full analytic sample used 248 observations for EDA SCL and 245 observations for EDA NS-SCR frequency.

^f^ The full analytic sample used 248 observations for EDA SCL and 245 observations for EDA NS-SCR frequency. Adjusted mixed-effects regression model with square root transformation.

**Table 3.** Secondary End Points

Measure	Mean (SD)		Complete pairs		Cohen d	All observations^a^		
	SADE	RDE	No.^b^	*P* value^c^		No.^d^	*P* value^e^	β (95% CI)
**Skin conductance level**									
Baseline	5.51 (3.87)	7.43 (4.79)	117	<.001	−0.41	266	<.001	−0.37 (−0.53 to −0.20)
Oral examination	7.01 (4.53)	8.06 (5.12)	100	.01	−0.26	238	.03	−0.20 (−0.39 to −0.02)
Prophylaxis	7.78 (4.89)	9.20 (5.35)	100	.01	−0.23	238	.01	−0.23 (−0.42 to −0.05)
Fluoride	7.67 (5.60)	9.71 (5.90)	94	.001	−0.29	228	.002	−0.34 (−0.55 to −0.13)
**Nonspecific skin conductance response frequency**									
Baseline	3.95 (2.93)	5.57 (3.39)	114	<.001	−0.55	263	<.001	−0.41 (−0.58 to −0.24)
Oral examination	3.63 (2.75)	4.10 (3.01)	100	.05	−0.19	236	.09	−0.13 (−0.28 to 0.02)
Prophylaxis	3.65 (2.95)	3.88 (3.05)	100	.44	−0.06	237	.34	−0.07 (−0.22 to 0.08)
Fluoride	4.26 (3.68)	4.62 (4.13)	94	.59	−0.06	226	.54	−0.08 (−0.33 to 0.17)
Pain^e^	2.14 (3.13)	2.37 (3.50)	57	.92	−0.01	144	.88	0.07 (−0.84 to 0.98)
Sensory discomfort^f^	3.06 (2.74)	3.68 (3.06)	53	.16	−0.11	133	.10	−0.53 (−1.16 to 0.10)
**Behavioral distress^f^**									
CDBRS	46.10 (10.77)	46.41 (11.78)	138	.87	−0.03	300	.95	−0.05 (−1.59 to 1.49)
Frankl Scale	2.68 (0.98)	2.61 (1.01)	138	.80	0.01	300	.61	−0.03 (−0.16 to 0.10)
Anxiety & Cooperation Scale	1.88 (1.61)	2.03 (1.77)	138	.76	−0.02	300	.49	0.07 (−0.14 to 0.29)
Head movement frequency	11.68 (13.92)	16.36 (19.39)	138	<.001	−1.19	277	<.001	−5.55 (−7.22 to −3.87)
Mouth movement frequency	3.20 (4.15)	5.43 (6.32)	138	<.001	−1.06	277	<.001	−2.49 (−3.38 to −1.61)
Whimper, cry, scream frequency	6.02 (7.10)	9.83 (9.96)	138	<.001	−1.29	279	<.001	−4.21 (−5.44 to −2.97)
Whimper, cry, scream duration	19.7 (43.53)	44.26 (68.39)	138	<.001	−0.84	279	<.001	−24.25 (−33.03 to −15.47)

Abbreviations: CDBRS, Children’s Dental Behavior Rating Scale; RDE, regular dental environment; SADE, sensory-adapted dental environment.

^a^ Mixed-effects regression model with adjustment for attained age and first or second clinic visit. EDA values were square-root transformed for running only the models including all observations; β values and 95% CIs represent the model estimates for the SADE − RDE values in terms of the square root transformations since a simple back transformation does not represent a difference in untransformed values.

^b^ Number of children with a nonmissing value for that variable for both dental visits.

^c^ Wilcoxon signed rank test.

^d^ Number of dental visits with a nonmissing value for that variable.

^e^ Comparing means and SDs for all observations, including incomplete pairs and missing data, using a mixed-effects regression model.

^f^ For all behavioral distress variables other than the Frankl Scale, higher scores reflect more challenging behavior; a lower score indicates more negative behavior on the Frankl Scale.

No significant differences were found between SADE and RDE for NS-SCR frequency (mean difference, −0.30 [95% CI, −0.86 to 0.25] per min) (Table 2). However, there was a significantly smaller frequency of NS-SCRs in the baseline component of the dental cleaning in the SADE condition (mean [SD], 3.95 [2.93] NS-SCRs per minute) compared with the RDE condition mean [SD], 5.57 [3.39] NS-SCRs per minute) (Table 3).

### Secondary Outcomes

Video-coded measures of distress behavior yielded mixed results. No differences between conditions were found using CDBRS total scores. However, analysis of frequency and duration of behavioral distress responses yielded significant differences in all variables scored, with participants exhibiting less frequency and duration of behavioral distress in SADE compared with RDE (Cohen *d* = −0.84 to −1.29) (Table 3). No significant differences were found from dentist-report of child behavior or child-report of pain or sensory discomfort (Table 3).

### Mediation Analysis

Video-coded frequency and duration of behavioral distress were associated with treatment condition, so the mediating effect of physiological stress on this association was assessed. For all video-coded frequency and duration behavioral distress outcomes, the β estimate for treatment effect was lower when physiological anxiety was added to the model, indicating that it was mediating some portion of the treatment effect. However, the treatment effect remained highly significant for each outcome, suggesting that physiological anxiety did not fully explain the difference in outcomes due to treatment (eTable 2 in Supplement 2). For frequency of head movement, mouth movement, and whimper, cry, or scream, NS-SCR frequency was associated with outcome independent of treatment and SCL, while NS-SCR frequency was not associated with whimper, cry, or scream duration independent of treatment and SCL. SCL was not associated with any of the outcomes independent of treatment and NS-SCR frequency.

Because the other questionnaire-based behavioral outcomes were not associated with treatment condition, we wanted to see whether the null association was due to a lack of association between behaviors and physiological stress. However, physiological stress and anxiety (measured via EDA), were found to be associated with behavioral distress and dental-related sensory discomfort (eTable 3 in Supplement 2). Specifically, dental cleaning SCL was significantly associated with behavioral distress (Anxiety and Cooperation scale: standardized β = 0.13 [95% CI, 0.03 to 0.24]; Frankl scale: β = −0.11 [95% CI, −0.22 to −0.01]). Dental cleaning NS-SCR frequency was likewise significantly associated with behavioral distress (Anxiety and Cooperation scale: β = 0.32 [95% CI, 0.21 to 0.42]; Frankl scale: β = −0.29 [95% CI, −0.39 to −0.19]; Children’s Dental Behavior Rating scale: β = 0.21 [95% CI, 0.10 to 0.32]; head movement frequency: β = 0.12 [95% CI, 0.03 to 0.21]; mouth movement frequency: β = 0.21 [95% CI, 0.10 to 0.32]; whimper, cry, or scream frequency: β = 0.12 [95% CI, 0.02 to 0.22]) and dental-related sensory discomfort (β = 0.18 [95% CI, 0.01 to 0.35]). In addition, baseline NS-SCR was associated with behavioral distress during the dental cleaning (Anxiety and Cooperation scale: β = 0.15 [95% CI, 0.04 to 0.25]; Children’s Dental Behavior Rating scale: β = 0.13 [95% CI, 0.02 to 0.24]; head movement frequency: β = 0.10 [95% CI, 0.02 to 0.19]; mouth movement frequency: β = 0.14 [95% CI, 0.03 to 0.25]) and sensory discomfort (β = 0.26 [95% CI, 0.09 to 0.42]).

### Moderation Analysis

No significant effect moderation on either SCL or NS-SCR frequency was found for any of the potential effect moderators (eTable 4 in Supplement 2). However, age, full-scale IQ, expressive communication, and sensory overresponsivity were significant effect moderators for behavioral distress, as measured by duration and frequency, suggesting that SADE was more efficacious in decreasing behavioral indicators of distress in younger children (ie, ages 6-9 years), as well as those with lower IQ, expressive communication, or sensory overresponsivity (eTable 5 in Supplement 2).

We considered that some moderation could be the result of confounding with baseline values for the outcome variables. However, including baseline values in the models changed results very minimally. Order of visits (RDE vs SADE first) was considered as a covariate, despite it not being a likely confounder as it was a randomized variable, with no significant findings (eTable 4 and eTable 5 in Supplement 2).

## Discussion

This randomized crossover study found that dental cleanings performed in SADE elicited significantly lower sympathetic activation, as measured by SCL, compared with RDE throughout the entirety of the dental encounter, suggesting that children were more relaxed and less physiologically anxious during care in SADE. This finding supports previous studies reporting the efficacy of SADE to decrease a variety of physiological measures of stress and anxiety in smaller sample sizes of autistic children,^26^ children with developmental disabilities,^23,24^ and adults with intellectual and developmental disabilities.^25^

Although SCL and NS-SCRs are widely used in research as indicators of sympathetic activation, it should be noted that they are only moderately positively correlated^33,37^ and may measure different sympathetic constructs, with some research suggesting that SCL is more responsive to fear while NS-SCRs increase more in anger.^33^ These nuanced differences in sympathetic arousal may explain why our results found significant decreases in SCL but not NS-SCR frequency when comparing conditions.

Children in this study also exhibited significantly decreased overt behavioral distress indicators, as measured by frequency and duration of distress behavior, in SADE compared with RDE. This finding supports previous research indicating a positive behavioral impact of SADE.^20,23,24,25,27^ As uncooperative behavior is commonly reported as a barrier to care for autistic children,^10,11,38^ use of SADE is a potential technique to support care and enhance successful experiences for this population.

Although we found significant improvements in frequency and duration of distress behavior, there were no significant changes in the CDBRS, Frankl Scale, or Anxiety and Cooperation Scale. We hypothesize this may be due to a lack of sensitivity of these measures to assess change. A random measurement error can cause results that cluster around the true value but which are imprecise. For example, the Frankl and Anxiety and Cooperation scales are 4- and 6-point Likert Scales, respectively, and the CDBRS codes only a dichotomous presence or absence for distress variables. Although Kim et al^20^ reported a significant decrease in uncooperative behavior for children with developmental disabilities in SADE compared with RDE using the Frankl Scale, they used a modified scoring rubric. The remaining studies that have reported behavior improvements in SADE vs RDE all used measurements of frequency, duration, or magnitude of distress behavior, similar to this study.^23,24,25^ Similarly, there were no significant changes in child perception of pain and sensory discomfort, which we also believe may be due to a lack of sensitivity (6- and 3-point scales, respectively).

EDA was significantly lower at baseline in SADE vs RDE, suggesting that simply lying in the sensory-adapted room prior to treatment elicited relaxation. EDA at baseline was also significantly associated with future sympathetic activation during the cleaning, overt distress behavior, and child-report of sensory discomfort. Therefore, these results support the potential impact of environmental factors on stress and relaxation immediately on entering the dental operatory and extending throughout the duration of the dental encounter.

Similar to the findings of Kim et al,^20^ no demographic variables of interest moderated the effect of SADE on physiological distress in our study, suggesting that SADE is equally efficacious in reducing sympathetic activation for autistic children regardless of demographic differences. However, as expected, age, IQ, and expressive communication were associated with moderating multiple measures of frequency and duration of behavioral distress; this is especially meaningful, since children with lower IQ or communication are less likely to respond to other approaches to reduce stress during medical procedures that rely more on verbal means. Although surprising, the absence of moderation due to child sensory overresponsivity, dental fear, or anxiety may be due to a lack of meaningful variation in these variables in our sample; perhaps once some minimal threshold of sensory overresponsivity, dental fear, or anxiety is met, SADE no longer differentially impacts care.

The SADE approach is highly scalable, as it requires minimal training to implement, is easily portable, does not involve renovations, requires only 5 to 10 minutes to set up or remove, and incurs a 1-time cost of less than $6000. In practice settings, this equipment could remain set up indefinitely, with clinicians able to use (or not use) SADE adaptations for any given patient. One of the greatest challenges with implementation of newly developed interventions is the translation of research into practice. Due to the simplicity and high scalability of SADE, it has outstanding potential to be readily implemented into dental clinics nationwide. In fact, SADE has recently been added to the American Academy of Pediatric Dentistry’s best practices for behavioral guidance as a potential technique for dental patients with anxiety or special health care needs.^39^ Future research should investigate the effectiveness of SADE to improve behavioral and physiological distress in autistic children in a multisite effectiveness trial, disentangle whether the individual components of SADE separately improve outcomes or whether SADE’s success is due to its application as a whole package; examine the possible long-term effects of SADE, determine the impact of SADE on the dental team, consider further tailoring of treatment based on child characteristics and interests, and identify other populations and medical procedures that may benefit from this approach.

### Limitations

This study has some limitations; however, implementing lessons learned from our pilot study enabled us to successfully avoid many in the execution of this study. First, despite our efforts to minimize the burden to study participation, our attrition rate was greater than anticipated, with 162 participants completing 1 dental cleaning and 138 participants completing 2, from the original 220 participants enrolled. Study features minimizing the potential effect of missing data included that participants with one dental cleaning were demographically similar to those with 2, any unmeasured participant characteristic associated with missing a dental visit would have been equally likely to have been randomized to SADE first as to RDE first, and use of statistical methods where data are missing completely at random is not required. Second, because our consecutive sampling strategy, our sex distribution reflects more closely the autism distribution reported in the community (1 girl to 4 boys). Future research should examine possible sex-specific differences for SADE, since there are documented health-related sex differences in the autistic population.^40^ Because this recruitment strategy only confirmed eligibility of those contacted by the team, we are unable to generalize results to potentially eligible participants who were not enrolled; this issue of generalizability could also apply to participants who attended only 1 dental cleaning. Third, due to the nature of our environmental modifications, it was not possible to blind all outcome measures. To address this, we ensured that at least 16% to 20% of videos were independently double-coded to improve accuracy and included blinded measures (EDA), which still yielded significant results. Fourth, although we believe a permanent SADE equipment set-up would not impact clinical workflow or turnover time between patients, this must be formally assessed in future research.

## Conclusions

This randomized crossover trial is the first large-scale study, to our knowledge, to examine the efficacy of a SADE to improve dental care for autistic children. Findings support the use of SADE to significantly improve both physiological and behavioral distress in this population during routine dental cleanings. Use of SADE is relatively inexpensive, scalable, and easy to implement with minimal training.

## Notes

Supplement 1.
Trial Protocol and Statistical Analysis Plan

Supplement 2.
eMethods.
**eTable 1.** Participant Characteristics by Number of Completed Dental Visits **eTable 2.** Physiological Anxiety as a Potential Mediator of the Association of SADE or RDE With Video-Coded Frequency and Duration of Distress Behaviors **eTable 3.** Effect of Physiological Stress on Primary and Secondary Outcomes **eTable 4.** Effect Moderation of Physiological Distress (EDA Values) by Subgroup **eTable 5.** Effect Moderation of Behavioral Distress (Video-Coded Values) by Subgroup
eReferences.

Supplement 3.
Data Sharing Statement
