# Supplementary material for: Sensory Adaptations to Improve Physiological and Behavioral Distress During Dental Visits in Autistic Children: A Randomized Crossover Trial
Source: JAMA Netw Open. 2023 Jun 2;6(6):e2316346. doi: 10.1001/jamanetworkopen.2023.16346 (PMC10238943; doi:10.1001/jamanetworkopen.2023.16346)
Supplement: Supplement 1. — Trial Protocol and Statistical Analysis Plan [file jamanetwopen-e2316346-s001.pdf]

STUDY PROTOCOL

TITLE
Sensory Adapted Dental Environments to Enhance Oral Care for Children (SADE-2 Study)

TRIAL REGISTRATION
ClinicalTrials.gov Identifier: NCT02430051
https://clinicaltrials.gov/ct2/show/NCT02430051?term=sensory+adapted+dental+environments&draw=2&rank=1

FUNDING
National Institute of Dental and Craniofacial Research (U01 DE024978)

PRINCIPAL INVESTIGATOR
Sharon Cermak
Chan Division of Occupational Science & Occupational Therapy
(323) 442-2879
cermak@usc.edu

BACKGROUND
This randomized clinical trial, entitled Sensory Adapted Dental Environments to Enhance Oral Care for Children (SADE-2 Study), is a follow up of our R34 pilot study, Sensory Adapted Dental Environments to Enhance Oral Care for Children with Autism (1R34DE022263-01). We propose to examine the efficacy of a sensory adapted dental environment to decrease physiological anxiety and negative responses (distress behavior, perception of pain, sensory discomfort) in children with autism spectrum disorders (ASD), many of whom have dental anxiety and/or sensory over-responsivity. Many of our subjects will have unmet dental needs, either from the conditions mentioned above or because our recruiting sources over-represent low income, Latino families who are reported to have greater challenges in accessing care.

Children with disabilities are almost twice as likely to suffer from unmet oral health care needs than their peers without disabilities,[1] and consequently have an increased risk and prevalence of dental disease.[2,3] Children with ASD, a condition characterized by impaired social-communication and social interaction, and restricted, repetitive patterns of behavior, interests or activities,[4] represent one such special population at high risk.[5,6] In fact, a large number of studies indicate that children with ASD exhibit a high incidence of poor oral health, as measured by caries prevalence and severity,[2,5-9] although there are a small number of contradictory studies.[10-12] The prevalence of ASD is significantly higher today than in the past, estimated in 2014 to be approximately 1 in 68 children in the US.[13] Therefore, dentists are increasingly likely to encounter children with ASD in their practices.

One factor that may contribute to oral care challenges in children with ASD is sensory over-responsivity,[14] characterized by behavioral responses that are out of proportion to the type or amount of stimulation. These responses may include physical

withdrawal, vocal outbursts, aggressive behaviors, tantrums, or attempts to block incoming stimulation.[15,16] Although the mechanisms underlying sensory processing disorders are not fully understood,[17-20] such problems are highly prevalent in the ASD population.[21-25] Up to 95% of children with ASD demonstrate significantly different sensory behaviors than their TD counterparts, and 61% specifically exhibit tactile over-responsivity symptoms as evidenced by difficulty tolerating grooming and hygiene tasks.[26]

Exacerbation of sensory over-responsivity in the dental office can result from exposure to bright lights, loud or high-pitched noises, reclining in the dental chair, repeated touch in and around the mouth, and the texture, taste, and smell of various oral care products. In a survey of 196 parents of children with ASD, up to 70% of parents reported that their child experienced difficulty with each of these sensory variables in the dental office, with the greatest difficulty being instruments in the mouth (70%).[27] In this study, almost 50% of parents of children with ASD strongly agreed that their child's sensory over-responsivity made visits to the dentist more challenging. Additionally, children with ASD with sensory over-responsivity, compared to children with ASD without sensory over-responsivity, exhibited a significantly greater prevalence of oral care difficulties in the home and dental office.[28] In focus groups that we conducted, parents reported reluctance to return to the dentist because of their child's negative experiences. For example, one parent stated that "The first time we took him to the dentist, when I heard him screaming for me from the front, I kind of understood how bad this was…there was like several people trying to restrain him. They had him in restraints and my wife [in the room with him] was in tears…because of that experience we were extremely hesitant to take him back."[29]

The proposed SADE-2 intervention modifies the sensory characteristics of the dental environment. It does so by altering the degree and type of visual, auditory, and tactile stimulation that children experience during dental treatment by reducing the lighting, providing soothing sounds, and applying deep pressure input to the child during a dental cleaning. Our hypothesis is that these changes will reduce physiological anxiety, negative behavior, pain, and sensory discomfort, and enhance cooperation to enable thorough and effective oral care.

The proposed study attempts to replicate and extend Shapiro et al.'s previous work[30-32] with children with developmental disabilities (not including ASD) in Israel, in which a sensory adapted dental environment resulted in shorter duration of negative behaviors and greater relaxation during treatment as measured by electrodermal activity (EDA), which assesses the sympathetic "fight or flight" system. Results from our R34 pilot study[33] examined differences in children's physiological responses in the two intervention conditions (sensory adapted environment, SADE, and a regular dental environment, RDE), finding that measures of electrodermal activity were lower (i.e. children were more relaxed) in the SADE. When examining the behavioral and survey measures, outcomes were in the hypothesized direction with primarily small effects (less uncooperative distress behavior, reduced perception of pain, and less sensory discomfort in the SADE vs. RDE). However, our R34 study was designed as a pilot study and therefore not powered to detect differences between the two dental environments. The preliminary positive benefit of the sensory adapted dental environment found in children

with ASD warrants a large-scale trial to appropriately power the study and enable examination of moderating and mediating variables.

In the proposed U01, we will test the effects of the SADE in a larger group of children with ASD to examine intervention efficacy and possible mediating and moderating factors. Our sample will be ethnically diverse, with more than 50% of the sample underserved, low income Latino children.

The development of a modified dental environment may help pediatric dentists, general dentists, and dental hygienists reduce common behavioral challenges that occur in treating children receiving treatment at the dental office. Because general dentists indicate that behavior problems are the greatest barrier treating children with disabilities,[34] decreasing children's distress behaviors may increase dentists' willingness to treat children with ASD and other children who are difficult to treat, thereby contributing to a reduction in health disparities. Treatment may also become more efficient and cost effective. If the child is more cooperative, the dentist may be able to complete a more thorough cleaning and better preventive care. Safety would also increase as the need for restraint (e.g., protective stabilization provided by a papoose board) and/or pharmacological intervention (e.g., nitrous oxide, general anesthesia) decreases. Research currently indicates that restraint is utilized with children with ASD 18-33% of the time, significantly more than with TD children (1%).[27,35] In a survey study conducted by our team, 18% of children with ASD without sensory over-responsivity required restraint often or almost always for dental care, compared to 38% of children with ASD with reported sensory over-responsivity.[36] Finally, as ease of cleanings improves, parents may be more likely to bring their child to the dentist for routine oral care, which would impact public health.

OBJECTIVES

Primary Objective: To compare children's physiological anxiety (EDA - primary outcome) during the dental cleaning in SADE vs. RDE.

Secondary Objective 2: To compare children's negative behavioral responses (behavioral distress, perception of pain, sensory discomfort - secondary outcomes) during dental cleanings in SADE vs. RDE.

Secondary Objective 3: To test the degree to which physiological anxiety mediates the SADE intervention's effects on children's negative behavioral responses.

Secondary Objective 4: To test the degree to which specific child characteristics (e.g., sensory over-responsivity, dental anxiety, ASD severity, IQ, age) moderate the SADE intervention's effects on children's physiological and negative behavioral responses during dental cleaning.

Secondary Objective 5: To compare the SADE intervention to the RDE on quality of care, cost-effectiveness, and potential cost savings of dental cleaning.

TRIAL DESIGN

This Phase II clinical trial utilized a randomized crossover design with two conditions at a single-site to test the efficacy of the SADE intervention protocol in reducing children's physiological anxiety and negative responses.

METHODS

Eligibility criteria: Child eligibility criteria were: (1) Spanish- or English-speaking, (2) 6-12 years, (3) at least one previous dental cleaning, and (4) confirmed autism diagnosis. Exclusionary criteria included: (1) current/scheduled orthodontic braces; (2) taking anticholinergic medication(s); (3) sibling of enrolled participant; (4) genetic, endocrine, or metabolic dysfunction (e.g., Down syndrome); (5) significant motor impairment (e.g., cerebral palsy); (6) significant oral condition (e.g., cleft palate); or (7) medical condition placing the child at increased risk in study (e.g., uncontrolled seizures).

Subject recruitment: Families will be recruited using a consecutive sampling strategy from an extensive network including health clinics (e.g., CHLA dental clinic), community service providers (e.g., developmental disability providers, resource fairs), therapy and behavioral clinics, patient referrals, parent support groups, social media, and the Los Angeles Unified School District.

Sample size determination: Original sample size calculations for this study determined that 165 children with two dental visits would be necessary for 80% power at a 2-sided alpha level of 0.05 to detect Cohen's d effect size differences of 0.22. Estimates for the effect sizes that would be realized ranged from 0.11-0.69 depending on the specific outcome in question, with an estimate of 0.44-0.46 for the primary outcome.

Randomization: Following confirmation of autism diagnosis (via administration of ADOS-2), participants will be randomly assigned to the order of treatment, receiving either the regular dental environment (RDE) condition or sensory adapted dental environment (SADE) condition for their first cleaning. Because we anticipate different rates of participation between sex (due to the distribution of autism diagnosis by sex)[13] and age, randomization will be stratified by gender and age (6.0-9.5 years and 9.6-12.11 years) so there is a balance in order of treatment between boys and girls and younger and older children in each order of environment. Randomization will be performed by our PhD biostatistician following a blocked randomization schema for each of the gender-age stratum. We anticipate that the largest stratum will be younger boys (~56% of the sample), followed by older boys (24%), younger girls (15%), and older girls (6%), thus block sizes will vary so that the study team cannot anticipate what the first treatment for a participant will be.

Study Procedures: *Visit One* – The Consent/Assent process will be completed. Immediately following the consent/assent, parent- and child-report (when appropriate) surveys will be completed to obtain demographic and descriptive information about the child. *Visit Two* – Children will come to CHLA to confirm autism diagnosis via the administration of the ADOS-2 test; child IQ will also be assessed. *Visits Three & Four* – Two dental cleanings will take place approximately 6 months apart, one in a regular dental environment and the other in a sensory adapted dental environment. Immediately prior to each dental cleaning, assessments of oral health and intraoral photographs will be completed by the dental professional, followed by the completion of a standardized dental cleaning (oral examination, prophylaxis, fluoride application). Video-recordings of child behavior and psychophysiological measures of distress (i.e., electrodermal activity) will be recorded continuously throughout both dental visits. Upon completion of the

dental cleaning, a second set of intraoral photographs will be collected and dentist-, parent-, and child-report assessments will be completed.

Intervention*:* Dental cleanings in the *control condition* (RDE) and *experimental condition* (SADE) will take place in a private room in the CHLA Dental Clinic and will each require approximately 15-45 minutes to complete, and will take place approximately six months apart. The SADE environment will be set up prior to the child's entrance into the dental room by a member of the research team. The specific modifications that comprise the SADE intervention include:

- Visual: All direct overhead fluorescent lighting and the regular dental overhead lamp will be turned off; dark colored "black-out" removable curtains will cover the windows in the private dental room. The dentist will wear a head-mounted dental lamp directed into the patient's mouth, reducing bright lights shining into the child's eyes. Slow moving visual color effects (Snoezelen) will shine onto the ceiling in the child's visual field.
- Auditory: Rhythmic music will be projected via a portable speaker system. Music will be either Dan Gibson's Exploring Nature with Music or similar options.
- Tactile/Deep Pressure: The tactile deep pressure stimulus will consist of a butterfly wrap (a modified version of the wrap developed in Israel),[30] weighted with a regular pediatric dental X-ray bib. The wrap fits around the dental chair and is made of a washable material. The "wings" of the butterfly wrap around the child from shoulder to ankles, providing a deep "hugging" pressure to produce a calming effect.

Outcome Measurements:

- Primary outcome: children's physiological stress and anxiety will be measured by electrodermal activity (EDA) throughout the dental cleaning. EDA will be measured using the BIOPAC Systems, Inc. MP150 System. This physiological response is caused by sympathetic nervous system activation, which increases during stressful or painful situations.[37] Sensors will be placed on the child's fingers and EDA recordings will be made immediately prior to and continuously throughout each cleaning. Two measures of tonic EDA will be collected: skin conductance level (SCL) and frequency of non-specific skin conductance responses (NS-SCR).
- Secondary outcomes: behavioral indicators of distress exhibited during the dental cleanings will be measured by researcher-videocoded assessments and by dentist-report (e.g., Children's Dental Behavior Rating Scale, Frankl Scale, Anxiety & Cooperation Scale); child-report of pain (Faces Pain Scale-Revised) and sensory discomfort (Dental Sensory Sensitivity Scale) will also be collected. Cost and quality of care will be assessed by number of restraining hands required, need for pharmacological methods, and scoring of intraoral photographs using a modified Plaque Index.
- Due to the practicalities of this study and the need to modify the dental environment for each patient, blinding of treatment condition for patients or dentists is not applicable. Due to the same environmental modifications, video-recordings cannot be blinded for scoring; however, a minimum of 15% of video-recordings will be double coded to at least 85% agreement by reliable and trained

researcher team members. Researcher-scoring of physiological data and dentist-scoring of intraoral photographs will be completed by a trained team member blinded to environment.

Statistical Analysis:
Descriptive statistics will be performed for all variables of interest to describe the participant population at baseline, including attrition rate and missing data, to compare those randomized to SADE first vs. those randomized to RDE first. To account for missing data, mixed effects regression models (based on full information maximum likelihood estimation) will be used. Primary outcomes: mean EDA scores will be calculated for each stage of the dental cleaning for each child at each dental visit. Various statistical methods will evaluate robustness of the results, including: paired sample t-tests, Wilcoxon signed rank tests, mixed effects regression, and mixed effects regression with transformation (if needed). Secondary outcomes: Wilcoxon signed rank tests and mixed effects regression with adjustment for attained age and first vs. second clinic visit will be conducted; EDA variables will utilize a logarithmic or square root transformation to improve normality of data if necessary. To assess the mediating effect of physiological stress on behavioral outcomes, we will first examine the bivariate relationships between EDA and behavioral outcomes. Then, the mediators will be included in mixed effects regression models including behavioral outcomes to determine if the effect of the treatment is attenuated in comparison to when the mediators are not included in the models. Stratified least squares means and standard errors by RDE/SADE and by potential effect modifiers, dichotomized at their medians or a clinically meaningful cut-point, will also be calculated. The cost-effectiveness ratio of the cost relative to the quality of care will be calculated; these models are paired and will involve paired t-tests or non-parametric corollary for continuous outcomes if the outcome is normal or non-normally distributed, respectively. For dichotomous outcomes (e.g., need for pharmacological methods), McNemar’s test will be used to examine differences in need for the paired observations.

Data Monitoring: Hard-copy collected data will be stored in a locked file cabinet. Paper documents will be entered into our secure and password-protected database by trained research assistants; data entries will be double-entered by a second trained research assistant, ensuring accuracy and quality control of data entry. Electronic data (video-recordings, electrodermal activity) will be transferred to a secure computer server in the USC Division of Occupational Science and Occupational Therapy that is only accessible to those with the correct security clearance and password; original files (e.g., video-recording stored on the camera) will then be deleted.

Clinical site monitoring will be conducted by the National Institute of Dental and Craniofacial Research, performed by a Clinical Research Operations and Management Support contractor. This will ensure that the rights of human subjects are protected, that the study is implemented in accordance with the protocol and/or other operating procedures, and that the quality and integrity of study data and data collection methods are maintained.

PROTECTION OF SUBJECTS

The investigator will ensure that this study is conducted in full conformity with the principles set forth in The Belmont Report: Ethical Principles and Guidelines for the Protection of Human Subjects of Research, as drafted by the US National Commission for the Protection of Human Subjects of Biomedical and Behavioral Research (April 18, 1979) and codified in 45 CFR Part 46 and/or the ICH E6; 62 Federal Regulations 25691 (1997).

Informed Consent/Assent: A consent form describing in detail the study procedures and risks will be given to the child participant's caregiver. Consent forms will be IRB-approved, and the caregiver will be required to read and review the document or have the document read to him or her. The research team member will explain the research study to the caregiver and answer any questions that may arise. The caregiver will sign the informed consent document prior to any study-related assessments or procedures, and they may withdraw consent at any time throughout the course of the study. The rights and welfare of the subjects will be protected by emphasizing to them that the quality of their clinical care will not be adversely affected if they decline to participate in this study.

Study assent forms will be used for children with autism when appropriate. The study will be verbally described to the child and the child subject will be provided with a simplified form (assent) providing the details of the study intervention, study procedures, and potential risks. In order to assure that the child understands, the investigator will ask the child to verbally summarize what they are going to do. The child will then sign and date the assent. For those subjects who are unable to give assent based on their cognitive abilities, the child will be informed about the trial to the extent compatible with their understanding.

Immediately following the consent (parental permission) process, explanations of both the California Bill of Rights and Health Insurance Portability and Accountability Act (HIPAA) documents will also take place; caregivers will sign the HIPAA document and be provided with copies of both forms.

Safety monitoring. Any unanticipated problems, adverse events, or serious adverse events that occur during the trial will be reported to the Institutional Review Board and the National Institute of Dental and Craniofacial Research (when appropriate).

Potential risks. This study will take the utmost precaution to ensure the welfare of the subjects. This study is a conceptual replication and extension of Shapiro et al.'s previous work with children with developmental disabilities (other than autism) in Israel in which no immediate or long-range adverse events were reported as a result of the study intervention.[30-32] This was verified through personal conversation with Dr. Shapiro who was a consultant on the R34 grant. Additionally, in our R34 SADE pilot study (n=44 children; n=22 ASD, n=22 typically developing),[33] there were no unanticipated problems or adverse events.

For the current study, risks from dental cleaning using SADE are similar to traditional dental visits (e.g., gingival bleeding, mild discomfort). Additional risks related to the study include the possibility of: (1) **Feeling uncomfortable answering some research-related survey questions or being videotaped**. Neither of these potential risks were a problem in the R34 SADE pilot study; however, if subjects or their parents feel

uncomfortable answering some questions they may choose to not answer that question. If subjects or their parents feel uncomfortable being video-recorded, they can ask the researchers to stop the recording. (2) **Skin Irritation.** We will be using the BIOPAC MP150 System from BIOPAC Systems, Inc. (Goleta, California) to collect physiological data (electrodermal activity) from human subjects. We will be using BIOPAC EL507 electrodes (silver-silver chloride electrodes with isotonic gel) placed on the tips of the second and third digits of the participant's non-dominant hand and secured with non-stick medical tape. Sensors will be attached using standard laboratory procedures; however, some participants may experience skin irritation from the conductance gel used in the sensors. This clears up quickly after the sensors and the gel are removed. We previously utilized this system in our R34 pilot study with no reports of injury, skin irritation, or other adverse reactions to the electrodes, gel, or tape. Additionally, BIOPAC Systems, Inc. has tested the MP150 System to applicable medical device standards, even though, strictly considered, the MP150 is not a medical device. The applicable standards for medical safety requirements are determined by IEC 60601-1 and the applicable standards for electromagnetic compatibility requirements are determined by IEC 60601-1-2 (Self-Declaration of Conformity, BIOPAC Systems, Inc., 2011; *http://www.biopac.com/Corporate.asp?Index=1*). (3) **EDA fear.** Children may also feel uncomfortable having electrodes applied to their fingers. We will minimize children's discomfort by showing the electrodes to the participants as part of a home visit prior to the dental cleaning and by providing the children with a social story that shows electrode application. (4) **Butterfly Wrap.** There is a possibility that the butterfly wrap could inadvertently induce anxiety in our child participants. We will address this concern by allowing the children (or caregiver) to request discontinuation of its use at any time during the dental cleaning (refusals and/or discontinuations will be documented).

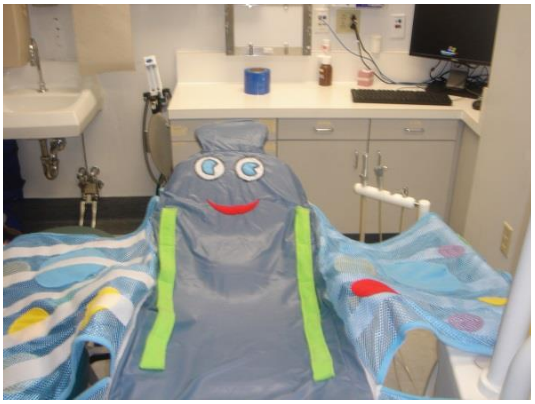

Potential benefits. Direct benefits for the child participant are two dental cleanings (oral exam, prophylaxis, and fluoride application) provided with no out-of-pocket costs. Additionally, scheduling both dental visits will occur with a minimal wait time (currently the dental clinic has a wait of approximately 8 or more months for scheduling a routine cleaning for a returning or new patient). Moreover, dental treatment in the SADE condition may prove more calming to children than in the regular dental environment. The potential indirect long-term benefits of this study include contributing to knowledge of factors that reduce physiological anxiety and negative responses during dental cleanings, and help children who are anxious or fearful at the dentist. Findings from this study may contribute to safer, more efficient, and less costly treatment for both children with autism as well as TD children with dental anxiety and/or sensory over-responsivity.

Withdrawal of Subjects. Subjects may withdraw voluntarily from the study. The investigator may also terminate a subject's participation if the subject meets an exclusion

criterion (either newly developed or not previously recognized), or if the second dental cleaning cannot be scheduled within timing requirements.

Compensation. Families will not incur any out-of-pocket costs for participating in this study (e.g., either the study will pay for both dental cleanings or participants' insurance will be billed for one cleaning and the other provided free of charge). In addition, participants will be paid a stipend following each visit to compensate families for their time as well as parking/transportation costs. Visit One – $40 following completion of study questionnaires. Visit Two – $40 following autism and IQ assessment + $10 parking/transportation. Visits Three & Four – $40 following each (attempted) dental cleaning + $10 parking/transportation.

References

1. Silver, E.J. & Stein, R.E.K. (2001). Access to care, unmet health needs, and poverty status among children with and without chronic conditions. *Ambulatory Pediatrics,1,* 314-320.
2. DeMattei, R., Cuvo, A., & Maurizio, S. (2007). Oral assessment of children with autism spectrum disorder. *Journal of Dental Hygiene, 81,* 65-76.
3. Seirawan, H., Schneiderman, J., Greene, V., & Mulligan, R. (2008). Interdisciplinary approach to oral health for persons with developmental disabilities. *Special Care in Dentistry, 28,* 43-52.
4. American Psychiatric Association (2013). *Diagnostic and statistical manual of mental disorders (5th ed.).*. Arlington, VA: American Psychiatric Publishing.
5. Kopycka-Kedzierawski, D.T., & Auinger, P. (2008). Dental needs and status of autistic children: Results from the national survey of children's health. *Pediatric Dentistry, 30,* 54-58.
6. Marshall, J., Sheller, B., & Mancl, L. (2010). Caries-risk assessment and caries status of children with autism. *Pediatric Dentistry, 32,* 69-75.
7. Child and Adolescent Health Measurement Initiative (2011). *National survey of children with special health care needs 200-10* [Online data set]. Retrieved from http://www.childhealthdata.org/browse/survey?s=1
8. Jaber, M. A. (2011). Dental caries experience, oral health status and treatment needs of dental patients with autism. *Journal of Applied Oral Science, 19*(3), 212-217.
9. Klein, U., & Nowak, A. (1999). Characteristics of patients with autistic disorder (AD) presenting for dental treatment: A survey and chart review. *Special Care in Dentistry, 19,* 200-207.
10. Fahlvik-Planefeldt, C. & Herrstrom, P. (2001). Dental care of autistic children within the non-specialized Public Dental Service. *Swedish Dental Journal, 25,* 113-118.
11. Loo, C.Y., Graham, R.M., and Hughes, C.V. (2008). The caries experience and behavior of dental patients with autism spectrum disorder. *Journal of the American Dental Association, 139,* 1518-1524.

12. Morinushi, T., Ueda, C., Tanaka, C., (2001). Autistic children: experience and severity of dental caries between 1980 and 1995 in Kagoshima City, Japan. *Journal of Clinical Pediatric Dentistry, 25*(4), 323-328.
13. Centers for Disease Control and Prevention (2014). Autism spectrum disorders: Data and statistics. Retrieved from http://www.cdc.gov/ncbddd/autism/data.html.
14. U.S. Department of Health and Human Services, National Institute of Dental and Craniofacial Research (2014). *Practical oral care for people with autism* (NIH Publication No. 04-5190). Retrieved from http://www.nidcr.nih.gov/OralHealth/Topics/DevelopmentalDisabilities/PracticalOralCarePeopleAutism.htm
15. Miller-Kuhaneck, H. (2008). Going to the Dentist. *Sensory Processing Disorder Foundation.* Retrieved from http://www.spdfoundation.net/library/dentist.html.
16. Tomcheck, S. D. (2010). Sensory processing in individuals with an autism spectrum disorder. *Autism: A comprehensive occupational therapy approach,* 135-161.
17. Bar-Shalita, T., Vatine, J., Seltzer, Z., & Parush, S. (2009). Psychophysical correlates in children with sensory modulation disorder (SMD). *Physiology and Behavior, 98,* 631-639.
18. McIntosh, D.N., Miller, L.J., Shyu, V., & Hagerman, R.J. (1999). Sensory-modulation disruption, electrodermal responses, and functional behaviors. *Developmental Medicine and Child Neurology, 41,* 608-615.
19. Rogers, S. J., & Ozonoff, S. (2005). Annotation: What do we know about sensory dysfunction in autism? A critical review of the empirical evidence. *Journal of Child Psychology and Psychiatry, 46,* 1255-1268.
20. Schoen, S. A., Miller, L. J., Brett-Green, B., & Hepburn, S. L. (2008a). Psychophysiology of children with autism spectrum disorder. *Research in Autism Spectrum Disorders, 2,* 417-429. doi:10.1016/j.rasd.2007.09.002.
21. Baker, A.E.Z., Lane, A., Angley, M.T., & Young, R.I. (2008). The relationship between sensory processing patterns and behavioural responsiveness in autistic disorder: A pilot study. *Journal of Autism and Developmental Disorders, 38,* 867-875.
22. Baranek, G.T., David, F.J., Poe, M.D., Stone, W.L., & Watson, L.R. (2006). Sensory experiences questionnaire: Discriminating sensory features in young children with autism, developmental delays, and typical development. *Journal of Child Psychology and Psychiatry, 47,* 591-601.
23. Baranek, G.T., Boyd, B.A., Poe, M.D., David, F.J., & Watson, L.R. (2007). Hyperresponsive sensory patterns in young children with autism, developmental delay, and typical development. *American Journal on Mental Retardation, 112,* 233-245.
24. Ben-Sasson, A., Cermak, S. A., Orsmond, G. I., Tager-Flusberg, H., Carter, A.

S., Kadlec, M. B., & Dunn, W. (2007a). Extreme sensory modulation behaviors in toddlers with autism spectrum disorders. *American Journal of Occupational Therapy, 61*, 584-592.

25. Ben-Sasson, A., Hen, L., Fluss, R., Cermak, S.A., Engel-Yeger, B., & Gal, E. (2009b). A meta-analysis of sensory modulation symptoms in individuals with autism spectrum disorders. *Journal of Autism and Developmental Disorders, 39,* 1-11.
26. Tomcheck, S.D. & Dunn, W. (2007). Sensory processing in children with and without autism: A comparative study using the short sensory profile. *American Journal of Occupational Therapy, 61,* 190-200.
27. Stein, L. I., Polido, J. C., Najera, S. O., & Cermak, S. A. (2012a). Oral care experiences and challenges in children with autism spectrum disorder. *Pediatric Dentistry, 34*, 387-391. doi: 10.5014/ajot.2012.004085.
28. Stein, L. I., Polido, J. C., & Cermak, S. A. (2012b). Oral care and sensory concerns in autism. *American Journal of Occupational Therapy*, 66, e73–e76. doi.org/10.5014/ajot.2012.00408.
29. Stein Duker, L.I., Henwood, B.F., Bluthenthal, R.N., Juhlin, E., Polido, J.C., & Cermak, S.A. (2017). Parents' perceptions of dental care challenges in male children with autism spectrum disorder: An initial qualitative exploration. *Research in Autism Spectrum Disorders, 39,* 63-72.
30. Shapiro, M., Melmed, R. N., Sgan-Cohen, H. D., Eli, I., & Parush, S. (2007). Behavioural and physiological effect of dental environment sensory adaptation on children's dental anxiety. *European Journal of Oral Science, 115,* 479-483. doi:10.1111/j.1600-0722.2007.00490.x
31. Shapiro, M., Sgan-Cohen, H.D., Parush, S., & Melmed, R.N. (2009a). Influence of adapted environment on the anxiety of medically treated children with developmental disabilities. *Journal of Pediatrics, 154,* 546-550.
32. Shapiro, M., Melmed, R.N., Sgan-Cohen, H., & Parush, S. (2009b). Effect of sensory adaptation on anxiety of children with developmental disabilities: A new approach. *Pediatric Dentistry, 31,* 222-228.
33. Cermak, S.A., Stein Duker, L.I., Williams, M.E., Dawson, M.E., Lane, C.J., & Polido, J.C. (2015). Sensory adapted dental environments to enhance oral care for children with autism spectrum disorders: A randomized controlled pilot study. *Journal of Autism and Developmental Disorders, 45,* 2876-2888.
34. Casamassimo, P.S., Seale, N.S., & Ruehs, K. (2004). General dentists' perceptions of educational and treatment issues affecting access to care for children with special health care needs. *Journal of Dental Education, 68,* 23-28.
35. Marshall, J., Sheller, B., Mancl, L., & Williams, B.J. (2008). Parental attitudes regarding behavior guidance of dental patients with autism. *Pediatric Dentistry, 30,* 400-407.

36. Stein, L. I., Polido, J., & Cermak, S. (2013). Oral care and sensory over-responsivity in children with autism spectrum disorder. *Pediatric Dentistry, 35*, 230-235.

37. Dawson, M. E., Schell, A. M., & Filion, D. L. (2007). The electrodermal system. In J. T. Cacioppo, L. G. Tassinary & G. G. Berntson (Eds.), *Handbook of psychophysiology* (3rd ed., pp. 159-181). New York: Cambridge University Press.
