# Supplementary material for: Sensory Adaptations to Improve Physiological and Behavioral Distress During Dental Visits in Autistic Children: A Randomized Crossover Trial
Source: JAMA Netw Open. 2023 Jun 2;6(6):e2316346. doi: 10.1001/jamanetworkopen.2023.16346 (PMC10238943; doi:10.1001/jamanetworkopen.2023.16346)
Supplement: Supplement 2. — eMethods. eTable 1. Participant Characteristics by Number of Completed Dental Visits eTable 2. Physiological Anxiety as a Potential Mediator of the Association of SADE or RDE With Video-Coded Frequency and Duration of Distress Behaviors eTable 3. Effect of Physiological Stress on Primary and Secondary Outcomes eTable 4. Effect Moderation of Physiological Distress (EDA Values) by Subgroup eTable 5. Effect Moderation of Behavioral Distress (Video-Coded Values) by Subgroup eReferences. [file jamanetwopen-e2316346-s002.pdf]

Supplemental Online Content

Stein Duker LI, Como DH, Jolette C, et al. Sensory adaptations to improve physiological and behavioral distress during dental visits in autistic children: a randomized crossover trial. *JAMA Netw Open*. 2023;6(6):e2316346. doi:10.1001/jamanetworkopen.2023.16346

eMethods.

eTable 1. Participant Characteristics by Number of Completed Dental Visits

eTable 2. Physiological Anxiety as a Potential Mediator of the Association of SADE or RDE With Video-Coded Frequency and Duration of Distress Behaviors

eTable 3. Effect of Physiological Stress on Primary and Secondary Outcomes

eTable 4. Effect Moderation of Physiological Distress (EDA Values) by Subgroup

eTable 5. Effect Moderation of Behavioral Distress (Video-Coded Values) by Subgroup

eReferences.

This supplemental material has been provided by the authors to give readers additional information about their work.

© 2023 Stein Duker LI et al. *JAMA Network Open.*

eMethods

Randomization Procedures

Following confirmation of ASD diagnosis (via administration of ADOS-2), participants were randomly assigned to the order of treatment, receiving either the regular dental environment (RDE) condition or sensory adapted dental environment (SADE) condition for their first cleaning. Because we anticipated different rates of participation between gender and age, randomization was stratified by gender and age (6.0-9.5 years and 9.6-12.11 years) so there was a balance in order of treatment between boys and girls and younger and older children in each order of environment (e.g., there were four groups: young boy, old boy, young girl, old girl). Randomization was performed by our PhD biostatistician using a SPSS random number generator with a uniform distribution following a blocked randomization schema for each of the gender-age stratum (blocks of eight for boy strata and four for girl strata given the expected higher number of boys than girls). For allocation concealment, the order of randomization was kept in a password protected excel document. There was one deviation from the randomization protocol in which one child accidentally received a dental cleaning in the same condition twice; as the objective of this study was to examine the change in response between conditions, the data from this participant was removed from the dataset (see Figure 1). In addition, a total of 12 participants withdrew or dropped out before the second visit for each randomization order (i.e., 12 participants for RDE→SADE and 12 participants for SADE→RDE) (see Figure 1).

Measures

Child Descriptor Measures.

Communication ability was measured through parent report on the expressive language subscale of the ***Vineland Adaptive Behavior Scales, Second Edition*** (VABS-II).[1] The tool has good reliability and validity and discriminates among autistic children with varying levels of severity.[1-3] Items were scored on a scale of 0-2 points (*0-never performed*, *1-sometimes/partially*, *2-usually*). A raw score of 83 is equivalent to the expressive language of a four-year-old; for the purposes of this study, a child's ability to adequately express their basic emotions (i.e., distress) during a dental visit was more meaningful than a standard score, therefore a dichotomous expressive communication variable was created (<83 vs. $\geq$83).

The ***Wechsler Abbreviated Scale of Intelligence, 2nd edition*** (WASI-II)[4] is a brief measure of cognitive ability with excellent reliability and validity[4,5] that contains four subtests which together provide a full-scale IQ score (FSIQ-4). An IQ score of 70 is used to determine intellectual disability, which was used as a clinically meaningful cut-off point for this study (FSIQ <70 vs. $\geq$70).

General anxiety was assessed using the ***Child and Adolescent Symptom Inventory-4 Autism Anxiety Scale*** (CASI-Anx)[6]. This 20-item scale is derived from the CASI-4R,[7] has satisfactory validity and reliability,[6,8] and is an appropriate measure for this population, regardless of the child's language or cognitive abilities.[9] This parent-reported measure is scored on a four-point scale (i.e., *0-never*, *1-sometimes*, *2-often*, *3-very often*) with greater anxiety indicated by higher scores.

Dental anxiety was assessed via the 15-item ***Children's Fear Survey Schedule – Dental Subscale*** (CFSS-DS).[10] The CFSS-DS can be self- or parent-report, and is scored on a 5-point Likert scale (i.e., *1-not afraid at all* to *5-very afraid*) with total scores 32-38 indicating borderline fear and those >38 suggesting high dental fear.[11] The psychometric properties of this assessment indicate that there is high reliability and validity for children.[11,12]

Sensory processing patterns and sensitivities were measured by parent-response on the ***SensOR Inventory***.[13] The SensOR Inventory is a 76-item questionnaire with dichotomous yes/no responses, consisting of six domains (i.e., tactile, auditory, visual, olfactory, food texture, movement-proprioceptive) that are summed for a total score. High internal consistency reliability was found for typically-responsive and over-responsive groups.[13]

The ***Autism Diagnostic Observation Schedule, 2nd edition*** (ADOS-2)[14] was administered by a research-certified team member in a private room at Children's Hospital Los Angeles. The ADOS-2 is a semi-structured, standardized performance-based observational assessment of social affect, communication, reciprocal social interaction, restricted and repetitive behaviors, imagination/creativity, stereotyped behaviors and restricted interests. A severity (comparison) score corresponding to level of autism-related symptoms (i.e., 3-4: low, 5-7: moderate, 8-10: high) was determined for each participant; scores below 3 (1-2: minimal to low severity) did not meet eligibility criteria for the study.

Outcome measures.

Overt behavioral distress

Dentist-report Measures. The Anxiety and Cooperation Scale[15] and Frankl Scale[16] were completed by the dentist immediately following each dental cleaning. Both tools utilize a one-item Likert Scale to assess patient

© 2023 Stein Duker LI et al. *JAMA Network Open.*

behavior; the Anxiety and Cooperation Scale ranges from 0 to 5, with a lower score indicating greater cooperation and higher score denoting greater distress, while the Frankl Scale ranges from 1-4 (1=definitely negative to 4=definitely positive) with a higher score indicating greater cooperation. Both tools have established reliability and validity.[17-19]

Pain intensity. Child-report of pain during the dental cleaning was collected immediately after the dental encounter using the *Faces Pain Scale–Revised*.[20] This scale consists of six faces which express distress from *no pain* (0) to *very much pain* (10) and has strong psychometric properties within pediatric populations.[21,22]

Sensory discomfort. Children reported how "bothered" they were by five different sensory stimuli (e.g., lights, sounds, smell, taste, movement) during the dental cleaning as well as the overall environment on the *Dental Sensory Sensitivity Scale*.[23] Each item was scored on a three-point Likert scale – no bother, a little bother, a lot of bother – and summed across items, with a higher score indicating more sensory discomfort during the dental cleaning.

Data Analysis

Power Analysis. Original sample size calculations for this study determined that 165 children with two dental visits would be necessary for 80% power at a 2-sided alpha level of 0.05 to detect Cohen's d effect size differences of 0.22. Estimates for the effect sizes that would be realized ranged from 0.11-0.69 depending on the specific outcome in question, with an estimate of 0.44-0.46 for the primary outcome. The number of children with two dental visits was only 138 in this study, which contributed to low statistical power for some outcomes, while realized effect sizes were sometimes higher and sometimes lower than expected.

Primary Outcome. Electrodermal activity was scored offline using the BIOPAC Acq*Knowledge* program and hand-checked for accuracy and artifact exclusion,[24,25] with 25% of data double-coded to ≥85% agreement. NS-SCRs with amplitudes ≥0.05μS were converted to a rate of fluctuations per minute. The EDA variables were not normally distributed and a square root transformation was superior to a log transformation in terms of normality.[24,26] All EDA mean values reported are in their untransformed condition for ease of comparison for readers who might be familiar with these measures. Statistical models that used square root transformed values have beta estimates that are consistent with the square root transformations. The square root transformations have been noted in all applicable tables.

All the linear mixed effects regression analyses used restricted maximum likelihood, an unstructured covariance structure, and only the intercept was random. These models were adjusted for attained age and first or second clinic visit only. In crossover studies, in the absence of dropouts or missing outcome data, demographic variables which do not change over time (such as sex and race) can be effect moderators but not confounders since they are not associated with exposure; therefore, they need not be included as covariates. In fact, this property is often mentioned as a particular advantage for crossover studies.[27] Although adjusting for demographic variables in crossover studies with missing data using mixed effects regression can minimally effect results, it can have no effect on the other statistical methods that we used to validate our results.

For the paired sample t-test and Wilcoxon signed-rank tests only children with data from both dental visits were included; the RDE score was subtracted from the SADE score for each child The square root transformation was not used for Wilcoxon tests since the transformation would not change the rank, nor for the t-tests (single sample of differences between SADE and RDE) since the treatment differences were reasonably normally distributed.

Although it is reasonable to assume that children will have more favorable dental visit experiences when they are older and after having a previous clinical experience, such carry over effects would be expected to have minimal effect on a trial where treatment order is randomized and thus have not been included in analyses.

Mediation analyses. Physiological stress measures were assessed for mediation of outcomes that were significantly associated with treatment conditions. First, a linear mixed effects regression models for each of the four video-assessed behavioral distress outcomes (head movement frequency, mouth movement frequency, whimper/cry/scream frequency, whimper/cry/scream duration) assessed the effect of the treatment condition on these outcomes (adjusted for attained age and first or second clinic visit). Second, the same four models were run, but this time including square root transformed values for average skin conductance level and average NS-SCR frequency as covariates in each model. Comparison of the beta estimates for the treatment condition variable in corresponding models indicates if the association between treatment condition and outcome is attenuated by the mediators.

Moderation analyses. These analyses used linear mixed effects regression to model each mean EDA for averages and for frequencies (primary outcomes), and each of the four video-coded values for distress behavior

© 2023 Stein Duker LI et al. *JAMA Network Open.*

frequency/duration as a function of attained age, first or second clinic visit, treatment condition, the potential effect moderator, and an interaction term of the treatment condition with the potential effect moderator. The interaction term indicates if the effect of the treatment condition (SADE vs. RDE) is different in one level of the potential effect moderator compared to the other.

© 2023 Stein Duker LI et al. *JAMA Network Open.*

eTable 1. Participant Characteristics by Number of Completed Dental Visits

	**Total**[a] N=162	**1 dental visit**[a] N=24	**2 dental visits**[a] N=138	**p-value**[b] (1 vs. 2 dental visits)
Age mean in yrs (SD)	9.16 (1.99)	8.55 (1.98)	9.26 (1.98)	0.11
First eligibility visit	9.16 (1.99)	8.55 (1.98)	9.26 (1.98)	0.11
First dental visit	9.65 (2.04)	9.10 (2.04)	9.75 (2.03)	0.15
Second dental visit	10.50 (2.07)	--	10.50 (2.07)	--
Treatment Order				
SADE first	80 (49.38)	12 (50)	68 (49.28)	1.00
RDE first	82 (50.62)	12 (50)	70 (50.72)	
Months between 1st and 2nd dental visit	8.96 (5.14)	--	8.96 (5.14)	--
Sex				
Male	136 (83.95)	22 (91.67)	114 (82.61)	0.37
Female	26 (16.05)	2 (8.33)	24 (17.39)	
Race				
Asian	13 (8.02)	1 (4.17)	12 (8.7)	
Black	14 (8.64)	3 (12.50)	11 (7.97)	
Native American	0	0	0	0.84
Pacific Islander	1 (0.62)	0	1 (0.72)	
White	124 (76.54)	19 (79.17)	105 (76.09)	
More than one	10 (6.17)	1 (4.17)	9 (6.52)	
Ethnicity				
Hispanic	117 (72.22)	20 (83.33)	97 (70.29)	0.22
Non-Hispanic	45 (27.78)	4 (16.67)	41 (29.71)	
Diagnoses				
ADHD	28 (17.28)	2 (8.33)	26 (18.84)	0.26
One or more other diagnoses[c]	67 (41.36)	13 (54.17)	54 (39.13)	0.18
ADOS-2[d] severity score mean (SD)	6.38 (1.79)	6.17 (1.49)	6.41 (1.84)	0.54
Low autism severity 3-4	33 (20.37)	5 (20.83)	28 (20.29)	
Moderate autism severity 5-7	94 (58.02)	15 (62.50)	79 (57.25)	0.83
High autism severity 8-10	35 (21.60)	4 (16.67)	31 (22.46)	
WASI-II FSIQ4[d] mean (SD)	72.35 (23.18)	67.67 (19.47)	73.16 (23.73)	0.29
<70	74 (48.05)	13 (54.17)	61 (46.92)	0.66
$\geq$70	80 (51.95)	11 (45.83)	69 (53.08)	
VABS-II Expressive Communication[d] mean (SD)	63.11 (28.05)	56.25 (27.06)	64.31 (28.15)	0.20
<83	106 (65.84)	19 (79.17)	87 (63.50)	0.17
$\geq$ 83	55 (34.16)	5 (20.83)	50 (36.50)	
SensOR Inventory Total mean (SD)	28.37 (14.00)	34.08 (15.48)	27.38 (13.54)	0.03
CASI-4 Autism Anxiety Scale[d] mean (SD)	20.20 (10.57)	21.65 (12.37)	19.95 (10.25)	0.48
CFSS-DS[d] mean (SD)	47.96 (14.19)	50.17 (17.13)	47.57 (13.66)	0.41
Mother's Education				
<HS	24 (14.81)	4 (16.67)	20 (14.49)	
HS	26 (16.05)	4 (16.67)	22 (15.94)	0.91
Some college or vocational	63 (38.89)	8 (33.33)	55 (39.86)	
College degree or more	49 (30.25)	8 (33.33)	41 (29.71)	
Father's Education				
<HS	26 (18.18)	5 (22.73)	21 (17.36)	
HS	44 (30.77)	8 (36.36)	36 (29.75)	0.55
Some college	37 (25.87)	6 (27.27)	31 (25.62)	
College degree or more	36 (25.17)	3 (13.64)	33 (27.27)	

© 2023 Stein Duker LI et al. *JAMA Network Open.*

[a]N (%) for categorical variables, unless marked as mean (SD) for continuous variables.
[b]Independent samples t-test for continuous variables; Fisher exact test for categorical variables.
[c]Other diagnoses available for selection on survey and number endorsed within the full analytic cohort of 162 children: learning disability (n=1), developmental disability (n=6), dyslexia (n=2), sensory integration (n=4), epilepsy (n=4), anxiety disorder (n=7).
[d]ADOS-2: Autism Diagnostic Observation Scale-2; WASI-II FSIQ4: Wechsler Abbreviated Scale of Intelligence Full Scale Intelligence Quotient (IQ score of 70 is used to determine intellectual disability); VABS-II: Vineland Adaptive Behavior Scales-II Expressive Communication (Raw score of 83 is equivalent to the expressive language of a four-year-old); CASI-4 Autism Anxiety Scale: Child and Adolescent Symptom Inventory, 4th edition, Autism Spectrum Disorder Anxiety Scale; CFSS-DS: Children's Fear Survey Schedule – Dental Subscale.

© 2023 Stein Duker LI et al. *JAMA Network Open.*

eTable 2. Physiological Anxiety as a Potential Mediator of the Association of SADE/RDE With Video-Coded Frequency and Duration of Distress Behaviors

Video-Assessed Outcome	Unadjusted for physiological anxiety[a]		Adjusted for physiological anxiety[b]	
	Beta estimate[c] (SE)	P-value	Beta estimate (SE)	P-value
Head Movement frequency	-5.55 (-7.22 to -3.87)	<0.001	-4.44 (-6.27 to -2.61)	<0.001
Mouth Movement frequency	-2.49 (-3.38 to -1.61)	<0.001	-2.24 (-3.13 to -1.36)	<0.001
Whimper/Cry/Scream frequency	-4.21 (-5.44 to -2.97)	<0.001	-2.98 (-4.11 to -1.84)	<0.001
Whimper/Cry/Scream duration	-24.25 (-33.03 to -15.47)	<0.001	-16.60 (-24.85 to -8.35)	<0.001

a. Mixed effects regression beta and p-values for outcome modeled as a function of SADE/RDE with adjustment for attained age and first or second clinic visit.
b. Mixed effects regression beta and p-values for outcome modeled as a function of SADE/RDE with adjustment for attained age, first or second clinic visit, and square root transformed values for average skin conductance level and average NS-SCR frequency.
c. Negative beta estimates represent lower values under SADE than RDE.

© 2023 Stein Duker LI et al. *JAMA Network Open.*

eTable 3. Effect of Physiological Stress on Primary and Secondary Outcomes

	Skin conductance level (SCL)		NS-SCR[a] frequency	
	**Baseline[b]**	**Dental cleaning[c]**	**Baseline[b]**	**Dental cleaning[c]**
Dental cleaning SCL	0.80 (0.72 to 0.88)***	--	0.46 (0.34 to 0.58)***	--
Dental cleaning NS-SCR frequency	0.24 (0.11 to 0.37)**	--	0.59 (0.48 to 0.69)***	--
Pain	0.04 (-0.15 to 0.23)	0.02 (-0.16 to 0.20)	0.05 (-.14 to 0.24)	0.04 (-0.13 to 0.21)
Sensory Discomfort	0.07 (-0.10-0.24)	0.09 (-0.08 to 0.26)	0.26 (0.09 to 0.42)**	0.18 (0.01 to 0.35)*
Anxiety & Cooperation Scale	0.08 (-0.03 to 0.18)	0.13 (0.03 to 0.24)**	0.15 (0.04 to 0.25)**	0.32 (0.21 to 0.42)***
Frankl Scale	-0.03 (-0.14 to 0.08)	-0.11 (-0.22 to -0.01)*	-0.08 (-0.19 to 0.02)	-0.29 (-0.39 to -0.19)***
Children's Dental Behavior Rating Scale	0.02 (-0.09 to 0.14)	0.07 (-0.04 to 0.18)	0.13 (0.02 to 0.24)*	0.21 (0.10 to 0.32)***
Head Movement frequency	0.02 (-0.07 to 0.11)	0.08 (-0.01 to 0.17)	0.10 (0.02 to 0.19)*	0.12 (0.03 to 0.21)**
Mouth Movement frequency	0.03 (-0.08 to 0.14)	0.07 (-0.04 to 0.18)	0.14 (0.03 to 0.25)**	0.21 (0.10 to 0.32)***
Whimper, Cry, Scream frequency	-0.01 (-0.12 to 0.09)	0.08 (-0.02 to 0.19)	0.06 (-0.04 to 0.16)	0.12 (0.02 to 0.22)*
Whimper, Cry, Scream duration	-0.04 (-0.15 to 0.07)	0.03 (-0.07 to 0.13)	0.06 (-0.05 to 0.16)	0.08 (-0.02 to 0.18)

Note. Table reports standardized beta (95% confidence interval) from a mixed effects regression model of outcome variable as a function of physiological stress measure adjusted for attained age, first or second clinic visit, and SADE vs. RDE. Positive beta values indicate that the physiological stress measure was positive related to the outcome measure.

a. NS-SCR = non-specific skin conductance responses.
b. Baseline = continuous 3-minute period immediately prior to the dental cleaning.
c. Dental cleaning = the combination of oral examination, prophylaxis, and fluoride application.
d. *p<0.05, **p≤0.01, ***p≤0.001

© 2023 Stein Duker LI et al. *JAMA Network Open.*

eTable 4. Effect Moderation of Physiological Distress (EDA Values) by Subgroup

	**EDA: SCL** (µS) mean (SE)[a]		**EDA: NS-SCR Frequency** (count per minute) mean (SE)[a]	
	**RDE**	**SADE**	**RDE**	**SADE**
Age[b]<9 years	8.21 (0.85)	6.94 (0.86)	4.38 (0.48)	3.98 (0.49)
Age[b]>9 years	9.97 (0.87)	8.84 (0.86)	4.28 (0.49)	4.04 (0.49)
p-value[3]	0.88		0.77	
Sex male	8.75 (0.48)	7.45 (0.49)	4.45 (0.28)	3.93 (0.28)
Sex female	10.60 (1.05)	9.89 (1.07)	3.73 (0.60)	4.44 (0.61)
p-value[c]	0.64		0.08	
FSIQ<70	9.94 (0.68)	9.30 (0.71)	4.84 (0.38)	4.96 (0.40)
FSIQ≥70	8.37 (0.59)	6.95 (0.58)	3.70 (0.33)	3.26 (0.33)
p-value[c]	0.43		0.31	
VABS<83	9.81 (0.56)	8.63 (0.59)	4.74 (0.32)	4.41 (0.33)
VABS>83	7.85 (0.72)	6.76 (0.69)	3.66 (0.40)	3.43 (0.39)
p-value[c]	0.93		0.86	
Autism severity 3-4	9.29 (0.95)	7.98 (0.97)	3.79 (0.53)	2.91 (0.54)
Autism severity 5-7	8.97 (0.58)	7.99 (0.60)	4.42 (0.33)	4.36 (0.33)
Autism severity 8-10	9.14 (1.04)	7.41 (1.03)	4.66 (0.59)	4.28 (0.59)
p-value[c]	0.83		0.47	
SensOR[d] <14	9.43 (1.10)	8.71 (1.19)	5.59 (0.61)	5.64 (0.66)
SensOR[d] >14	9.00 (0.49)	7.73 (0.49)	4.08 (0.27)	3.73 (0.27)
p-value[c]	0.68		0.59	
CFSS-DS parent<32	7.80 (1.23)	8.17 (1.20)	4.88 (0.69)	4.07 (0.68)
CFSS-DS parent>32	9.27 (0.48)	7.80 (0.49)	4.24 (0.27)	4.01 (0.28)
p-value[c]	0.17		0.45	
Pre-Covid	8.93 (0.49)	7.77 (1.21)	4.34 (0.28)	4.05 (0.28)
Post-Covid	9.80 (1.08)	7.86 (0.49)	4.27 (0.61)	3.80 (0.69)
p-value[c]	0.51		0.83	
Visit 3	4.27 (0.35)	3.83 (0.36)	9.25 (0.61)	7.33 (0.64)
Visit 4	4.40 (0.37)	4.22 (0.37)	8.89 (0.65)	8.44 (0.64)
p-value[c]	0.76		0.34	

Note. EDA=electrodermal activity; SCL=skin conductance level; NS-SCR: non-specific skin conductance responses; RDE=regular dental environment; SADE=sensory adapted dental environment; FSIQ=Wechsler Abbreviated Scale of Intelligence Full Scale Intelligence Quotient (IQ score of 70 is used to determine intellectual disability); VABS= Vineland Adaptive Behavior Scales-II (raw score of 83 is equivalent to the expressive language of a four-year-old); CFSS-DS=Children's Fear Survey Schedule-Dental Subscale (score >32 indicates borderline or clinical dental fear).

a. Least squares mean (standard error) with adjustment for attained age and visit; adjusted means calculated at attained age and visit means.
b. Age at baseline
c. P-value for interaction of subgroup with treatment (SADE/RDE), i.e., difference in treatment effect by subgroup.
d. Cut-score based on Schoen et al.[13]

© 2023 Stein Duker LI et al. *JAMA Network Open.*

eTable 5. Effect Moderation of Behavioral Distress (Video-Coded Values) by Subgroup

	**Head Movement Frequency** (count per first 5 mins of prophylaxis) mean (SE)[a]		**Mouth Movement Frequency** (count per first 5 mins of prophylaxis) mean (SE)[a]		**Whimper, Cry, Scream Frequency** (count per first 5 mins of prophylaxis) mean (SE)[a]		**Whimper, Cry, Scream Duration** (seconds per first 5 mins of prophylaxis) mean (SE)[a]	
	**RDE**	**SADE**	**RDE**	**SADE**	**RDE**	**SADE**	**RDE**	**SADE**
Age[b]<9 years	20.39 (2.58)	13.87 (2.58)	7.76 (0.84)	4.52 (0.83)	11.27 (1.37)	5.59 (1.36)	48.57 (9.04)	15.50 (8.96)
Age[b]>9 years	13.42 (2.60)	8.79 (2.61)	3.48 (0.84)	1.69 (0.85)	9.13 (1.37)	6.34 (1.38)	40.68 (9.05)	25.12 (9.09)
p-value[c]	0.27		0.10		0.02		0.05	
Sex male	17.71 (1.46)	11.41 (1.46)	5.71 (0.48)	3.13 (0.48)	9.86 (0.76)	5.54 (0.76)	45.55 (5.05)	19.28 (5.07)
Sex female	12.78 (3.36)	10.91 (3.36)	5.10 (1.09)	3.05 (1.09)	11.76 (1.70)	8.07 (1.70)	39.49 (11.31)	24.91 (11.31)
p-value[c]	0.05		0.66		0.71		0.32	
FSIQ<70	25.55 (1.67)	17.64 (1.66)	8.00 (0.62)	4.77 (0.61)	13.45 (0.95)	8.02 (0.94)	66.72 (6.01)	26.84 (5.92)
FSIQ>70	7.53 (1.59)	4.81 (1.61)	3.33 (0.58)	1.52 (0.59)	6.93 (0.90)	4.00 (0.92)	19.49 (568)	9.33 (5.81)
p-value[c]	0.002		0.12		0.03		0.001	
VABS<83	22.51 (1.53)	14.55 (1.54)	7.33 (0.51)	4.11 (0.51)	12.17 (0.85)	6.48 (0.85)	56.24 (5.38)	23.66 (5.41)
VABS>83	6.11 (2.11)	4.68 (2.11)	2.33 (0.69)	1.17 (0.69)	6.55 (1.17)	4.95 (1.17)	18.08 (7.39)	14.05 (7.39)
p-value[c]	<0.001		0.03		0.002		0.001	
Autism severity 3-4	4.71 (2.82)	2.64 (2.89)	2.28 (0.92)	1.18 (0.96)	6.79 (1.51)	2.59 (1.57)	10.19 (9.86)	1.24 (10.32)
Autism severity 5-7	19.32 (1.67)	12.08 (1.66)	5.89 (0.55)	3.14 (0.55)	10.69 (0.91)	6.29 (0.90)	51.01 (5.93)	21.48 (5.86)
Autism severity 8-10	22.24 (2.78)	17.60 (2.78)	8.11 (0.91)	4.87 (0.91)	11.93 (1.47)	8.07 (1.48)	60.47 (9.62)	33.90 (9.63)
p-value[c]	0.05		0.26		0.94		0.20	
SensOR[d]<14	26.19 (3.47)	15.20 (3.44)	6.48 (1.14)	3.51 (1.12)	12.21 (1.82)	8.39 (1.79)	69.59 (12.00)	32.34 (11.79)
SensOR[d]>14	15.31 (1.43)	10.78 (1.43)	5.46 (0.47)	3.05 (0.48)	9.82 (0.75)	5.54 (0.75)	40.21 (4.95)	18.27 (4.98)
p-value[c]	0.005		0.65		0.79		0.21	
CFSS-DS parent<32	9.30 (3.95)	6.89 (3.95)	2.43 (1.28)	1.25 (1.28)	5.98 (2.06)	4.79 (2.05)	14.18 (13.62)	8.53 (13.61)
CFSS-DS parent>32	17.89 (1.41)	11.92 (1.41)	6.02 (0.46)	3.36 (0.46)	10.70 (0.73)	6.10 (0.74)	48.41 (4.85)	21.69 (4.87)
p-value[c]	0.19		0.30		0.09		0.14	
Pre-Covid	17.20 (1.49)	11.43 (1.49)	5.57 (0.48)	3.00 (0.48)	9.99 (0.76)	5.54 (0.76)	45.42 (5.08)	20.55 (5.08)
Post-Covid	15.20 (3.12)	11.74 (3.18)	5.65 (1.08)	3.81 (1.11)	10.41 (1.69)	8.35 (1.74)	39.58 (11.40)	20.14 (11.71)
p-value[c]	0.43		0.60		0.24		0.70	
Visit 3	13.39 (1.88)	12.66 (1.85)	4.89 (0.61)	3.56 (0.60)	9.15 (0.97)	7.02 (0.95)	40.87 (6.48)	19.53 (6.33)
Visit 4	20.58 (1.90)	10.09 (1.94)	6.38 (0.63)	2.63 (0.65)	11.18 (0.99)	4.77 (1.03)	48.54 (6.65)	21.17 (6.87)
p-value[c]	0.05		0.11		0.09		0.71	

Note. RDE=regular dental environment; SADE=sensory adapted dental environment; FSIQ=Wechsler Abbreviated Scale of Intelligence Full Scale Intelligence Quotient (IQ score of 70 is used to determine intellectual disability); VABS= Vineland Adaptive Behavior Scales-II (raw score of 83 is equivalent to the expressive language of a four-year-old); CFSS-DS=Children's Fear Survey Schedule-Dental Subscale (score >32 indicates borderline or clinical dental fear).

© 2023 Stein Duker LI et al. *JAMA Network Open.*

a. Least squares mean (standard error) with adjustment for attained age and visit; adjusted means calculated at attained age and visit means.
b. P-value for interaction of subgroup with treatment (SADE/RDE), i.e., difference in treatment effect by subgroup.
c. Age at baseline
d. Cut-score based on Schoen et al.[13]

© 2023 Stein Duker LI et al. *JAMA Network Open.*

eReferences

1. Sparrow SS, Cicchetti DV, Balla DA. *Vineland Adaptive Behavior Scales.* 2nd ed. American Guidance Service; 2005.
2. Klin A, Saulnier CA, Sparrow SS, Cicchetti DV, Volkmar FR, Lord C. Social and communication abilities and disabilities in higher functioning individuals with autism spectrum disorders: The Vineland and the ADOS. *J Autism Dev Disord*. 2007;37(4):748–759. https://doi.org/10.1007/s10803-006-0229-4
3. Perry A, Flanagan HE, Dunn Geier J, Freeman NL. Brief report: The Vineland Adaptive Behavior Scales in young children with autism spectrum disorders at different cognitive levels. *J Autism Dev Disord.* 2009;39(7):1066-1078. doi:10.1007/s10803-009-0704-9
4. Wechsler D. *Wechsler Abbreviated Scale of Intelligence*. 2nd ed. Pearson; 2011.
5. McCrimmon AW, Smith AD. Review of the Wechsler Abbreviated Scale of Intelligence, Second Edition (WASI-II). J Psychoeduc Assess. 2013;31(3):337-341. https://doi.org/10.1177/0734282912467756
6. Sukhodolsky DG, Scahill L, Gadow KD, et al. Parent-rated anxiety symptoms in children with pervasive developmental disorders: Frequency and association with core autism symptoms and cognitive functioning. J Abnorm Child Psychol. 2008;36(1):117–128. doi:10.1007/s10802-007-9165
7. Gadow KD, Sprafkin J. *Child and Adolescent Symptom Inventory-4R*. Checkmate Plus; 2005.
8. Sprafkin J, Gadow KD, Salisbury H, Schneider J, Loney J. Further evidence of reliability and validity of the Child Symptom Inventory-4: parent checklist in clinically referred boys. *J Clin Child Adolesc Psychol*. 2022;31(4):513-524. doi:10.1207/S15374424JCCP3104_10
9. Williams MW, Stein Duker LI, Vigen CLP, Wylde C, Cermak SA. (2022). Brief report: Caregiver confidence in reporting anxiety symptoms in children with autism spectrum disorder. *J Autism Dev Disord.* 2022;52(8):3727-3733. doi: 10.1007/s10803-021-05210-6
10. Cuthbert MI, Melamed BG. A screening device: children at risk for dental fears and management problems. *ASDC J Dent Child*. 1982;49(6):432–436.
11. Ten Berge M, Veerkamp JS, Hoogstraten J, Prins PJ. Childhood dental fear in the Netherlands: prevalence and normative data. *Community dentistry and oral epidemiology*. 2022;30(2):101-107. https://doi.org/10.1034/j.1600-0528.2002.300203.x
12. Lopes D, Arnrup K, Robertson A, Lundgren J. Validating the dental subscale of the children's fear survey schedule using Rasch analysis. *Eur J Oral Sci*. 2013;121(3pt2):277-282. https://doi.org/10.1111/eos.12040
13. Schoen SA, Miller LJ, Green KE. Pilot study of the Sensory Over-Responsivity Scales: Assessment and Inventory. *Am J Occup Ther.* 2008;62(4):393-406. doi: 10.5014/ajot.62.4.393
14. Lord C, Rutter M, DiLavore PC, Risi S, Gotham K, Bishop S. Autism Diagnostic Observation Schedule. 2nd ed. Western Psychological Services; 2012.
15. Veerkamp JSJ, Gruythuysen RJM, van Amerongen WE, Hoogstraten J, Weerheijm K. Dentist's ratings of child dental-patients' anxiety. *Community Dent Oral Epidemiol*. 1995;23(6):356–359. doi: 10.1111/j.1600-0528.1995.tb00262.x
16. Frankl SN, Shiere FR, Fogels HR. Should the parent remain with the child in the dental operatory. *J Dent Child*. 1962;29:150–163.
17. Schriks MCM, van Amerongen WE. A traumatic perspectives of ART: Psychological and physiological aspects of treatment with and without rotary instruments. *Community Dent Oral Epidemiol*. 2003;31(1):15–20. doi: 10.1034/j.1600-0528.2003.00021.x
18. Versloot J, Veerkamp JSJ, Hoogstraten J. Dental anxiety and psychological functioning in children: Its relationship with behaviour during treatment. *Eur Arch Paediatr Dent*. 2008;9(Suppl 1):36–40. doi: 10.1007/BF03262654
19. Aartman IHA, van Everdingen T, Hoogstraten J, Schuurs AHB. Appraisal of behavioral measurement techniques for assessing dental anxiety and fear in children: A review. *J Psychopathol Behav Assess*. 1996;18:153–171.
20. Hicks CL, von Baeyer CL, Spafford PA, van Korlaar I, Goodenough B. The Faces Pain Scale-Revised: Toward a common metric in pediatric pain measurement. *Pain*. 2001;93(2):173–183. doi: 10.1016/S0304-3959(01)00314-1
21. Le May, Ballard A, Khadra C, et al. Comparison of the psychometric properties of 3 pain scales used in the pediatric emergency department: Visual Analogue Scale, Faces Pain Scale-Revised, and Colour Analogue Scale. *Pain*. 2018;159(8):1508–1517. doi: 10.1097/j.pain.0000000000001236

© 2023 Stein Duker LI et al. *JAMA Network Open.*

22. Naegeli, Heath L, Zhou C, Gupta N, Dampier C. Psychometric properties of a modified version of the Faces Pain Scale-Revised (Modified FPS-R) to evaluate worst pain in children and adolescents with Sickle Cell Anemia. *J Pediatr Hematol Oncol*. 2018;40(7), 499–503. https://doi.org/10.1097/MPH.0000000000001250
23. Cermak SA, Stein Duker LI, Williams ME, Dawson ME, Lane CJ, Polido JC. Sensory adapted dental environments to enhance oral care for children with autism spectrum disorders: A randomized controlled pilot study. *J Autism Dev Disord*. 2015;45(9):2876-2888.
24. Boucsein W, Fowles DC, Grimnes S, et al. (2012). Committee Report: Publication recommendations for electrodermal measurements. *Psychophysiology*. 2012;49(8):1017-1034. doi: 10.1111/j.1469-8986.2012.01384.x
25. Boucsein W. *Electrodermal activity*. 2nd ed. Springer; 2012.
26. Dawson ME, Schell AM, Filion DL. Chapter 10: The electrodermal system. In: Cacioppo JT, Tassinary LG, Berntson GG, eds. *Handbook of Psychophysiology*. 4th ed. Cambridge University Press; 2016:217-243. doi: 10.1017/9781107415782.010
27. Mills EJ, Chan AW, Wu P, Vail A, Guyatt GH, Altman DG. Design, analysis, and presentation of crossover trials. *Trials*. 2009;10(27), 1-6.

© 2023 Stein Duker LI et al. *JAMA Network Open.*
