# Supplementary material for: Sensory Adaptations to Improve Physiological and Behavioral Distress During Dental Visits in Autistic Children: A Randomized Crossover Trial
Source: JAMA Netw Open. 2023 Jun 2;6(6):e2316346. doi: 10.1001/jamanetworkopen.2023.16346 (PMC10238943; doi:10.1001/jamanetworkopen.2023.16346)
Supplement: Supplement 3. — Data Sharing Statement [file jamanetwopen-e2316346-s003.pdf]

Data Sharing Statement

Stein Duker. Sensory Adaptations to Improve Physiological and Behavioral Distress During Dental Visits in Autistic Children. *JAMA Netw Open*. Published June 02, 2023. doi:10.1001/jamanetworkopen.2023.16346

Data
Data available: Yes
Data types: Deidentified participant data
How to access data: Deidentified data and a data dictionary will be provided to interested parties after the publications and research presentations that address the specific aims and major secondary analyses of the study are completed. Contact lstein@chan.usc.edu
When available: beginning date: 06-01-2024

Supporting Documents
Document types: None

Additional Information
Who can access the data: Researchers whose proposed use of the data has been approved.
Types of analyses: Any purpose.
Mechanisms of data availability: After approval of a proposal.
